# A Dose–Response Study on the Relationship Between Red Meat Intake and Metabolic Dysfunction-Associated Steatotic Liver Disease (MASLD) in Southern Italy: Results from the Nutrihep Study

**DOI:** 10.3390/nu18061002

**Published:** 2026-03-21

**Authors:** Davide Guido, Manuela Siani, Maria Noemy Pastore, Gianluigi Giannelli, Giovanni De Pergola

**Affiliations:** 1 Unit of Data Science, National Institute of Gastroenterology “Saverio de Bellis”, IRCCS Hospital, Castellana Grotte, 70013 Bari, Italy; davide.guido@irccsdebellis.it (D.G.); manuela.siani@irccsdebellis.it (M.S.); 2 Scientific Direction, National Institute of Gastroenterology “Saverio de Bellis”, IRCCS Hospital, Castellana Grotte, 70013 Bari, Italy; gianluigi.giannelli@irccsdebellis.it; 3 Center of Nutrition for the Research and the Care of Obesity and Metabolic Diseases, National Institute of Gastroenterology IRCCS “Saverio de Bellis”, Castellana Grotte, 70013 Bari, Italy

**Keywords:** MASLD, red meat intake, survey, dose–response modeling, logistic regression, DAG

## Abstract

(1) **Background**: Metabolic dysfunction-associated liver disease (MASLD) has emerged as a leading cause of liver conditions globally. The increasing trend in meat consumption, particularly red meat, has prompted examination of its effects on cardiometabolic health. This study aimed to explore how varying levels of red meat intake relate to MASLD in a population from Southern Italy. (2) **Methods**: We analyzed data from a cross-sectional study involving 1192 participants (42.7% male), with complete data available from the second NUTRIHEP survey wave (2014–2016). Statistical analysis utilized adjusted dose–response modeling. (3) **Results**: Subjects with MASLD numbered 587 (49.2%), including 278 males (54.6%) and 309 females (45.2%). Red meat consumption between 75 and 90 g/day revealed an unfavorable influence on MASLD in males. Interestingly, sex seem to play a role in this association, both in harmful (OR > 1) and protective (OR < 1) ways, associated with specific foods such as liver (OR = 0.936, *p* = 0.087) and red meatballs (OR = 0.584, *p* = 0.023) in males and roast red meat (OR = 2.152, *p* = 0.097), red cutlet (OR = 0.540, *p* = 0.087), and red meat slices (OR = 0.952, *p* = 0.076) in females. (4) **Conclusions**: A suspicious dose–response relationship was observed solely in men, limited to intake levels between 75 and 90 g/day. Overall, red meat consumption did not exhibit a consistent dose–response trend with MASLD. Furthermore, preferences for specific types, cuts, and preparations of red meat were differentially associated with metabolic outcomes based on sex.

## 1. Introduction

Metabolic dysfunction-associated steatotic liver disease (MASLD) is currently the leading cause of chronic liver disease worldwide. In 2023, a multisociety statement proposed replacing the term NAFLD (Non-Alcoholic Fatty Liver Disease) with MASLD, defined as the presence of hepatic steatosis accompanied by at least one cardiometabolic risk factor, in the absence of excessive alcohol consumption [1]. MASLD is characterized by the accumulation of triglycerides within hepatocytes and is closely associated with obesity, insulin resistance, dyslipidemia, T2DM, and hypertension. It is therefore regarded as the hepatic manifestation of metabolic syndrome [2].

Over the past two decades, the prevalence of MASLD has increased markedly, reaching rates ranging from 15% to 30%, divergences that may reflect differences in the methods, diagnostic criteria, and case definitions employed [3]. This growing trend highlights MASLD as a significant global health threat and an increasing challenge for public health systems [4].

In the absence of any treatment, the disease progresses insidiously: over time, MASLD may evolve into metabolic dysfunction-associated steatohepatitis (MASH), characterized by hepatic steatosis with apoptosis, inflammation, and fibrosis. Without intervention, this condition may progress to advanced forms of liver injury, such as cirrhosis and hepatocellular carcinoma. In the past decade, MASLD has become the leading indication for liver transplantation in adults [5].

The pathogenesis of MASLD is complex and multifactorial, involving interactions between non-modifiable genetic factors and lifestyle-related environmental influences. Environmental factors, including physical inactivity and unhealthy dietary habits, significantly contribute to the development of metabolic syndrome and, consequently, MASLD. An unhealthy diet is the main modifiable factor contributing to MASLD [6] and is the key focus for its regression, especially given the current lack of effective pharmacological treatments [7,8].

Current EASL–EASD–EASO clinical practice guidelines recommend the Mediterranean Diet (Med Diet) as the dietary pattern of choice [9]. The Mediterranean model limits the consumption of red and processed meats, refined carbohydrates, simple sugars, and ultra-processed foods, whose dietary patterns have been shown to have detrimental effects on liver health [10].

Red meat refers to fresh, unprocessed skeletal muscle meat from mammals, including beef, veal, pork, lamb, mutton, horse, or goat, which is usually consumed cooked [11]. Although red meat has played a crucial role in human evolution as a major source of essential nutrients (including vitamins A, B6, D, and B12; folates; omega-3 polyunsaturated fatty acids; conjugated linoleic acid; minerals; and high-quality proteins), its growing consumption, particularly in Western countries, has raised significant metabolic health concerns [12]. Red meat is rich in saturated fatty acids (SFAs) and cholesterol, which are linked to insulin resistance, abdominal obesity, and metabolic syndrome, all key factors in the development of MASLD. Moreover, the heme iron it contains has been implicated in the generation of reactive oxygen species (ROS), the promotion of inflammation, and alterations in glucose metabolism [13].

Cooking red meat also results in the formation of advanced glycation end-products (AGEs), which are pro-inflammatory and pro-oxidative molecules that contribute to the onset of insulin resistance by negatively affecting intracellular signaling pathways [14]. Several studies have linked dietary patterns to NAFLD and liver fibrosis. In a large case–control study in Southern India, the consumption of red meat, animal fat, nuts, and refined rice was positively associated with both NAFLD and the presence of fibrosis [15]. In a study conducted in Iran involving 1612 participants enrolled in the Golestan Cohort Study, who typically had a low intake of red meat, those in the highest quartile of consumption demonstrated a significantly increased likelihood of developing NAFLD [16]. Similarly, a prospective study using UK Biobank data found that higher red meat intake, along with lower consumption of fruit, cereals, tea, and dietary fiber, was associated with an elevated risk of NAFLD, cirrhosis, and liver cancer [17]. Additionally, Amina et al. found a significant association between red meat consumption and MASLD and fibrosis, as well as T2DM and hypercholesterolemia in the UK population [18]. Another prospective study of 316 participants at a medical center in Tel Aviv reported that both red meat consumption and changes in intake over time were linked to NAFLD and liver fibrosis [19]. A recent systematic review and dose–response meta-analysis revealed a potential positive association between red meat consumption and the risk of NAFLD. However, it remains unclear whether the effect of unprocessed red meat is as significant as that of processed red meat [20].

The present study aims to further explore the association between red meat and MASLD and contribute additional evidence to clarify remaining uncertainties.

To date, no dose–response investigations have examined the relationship between red meat consumption and MASLD in a Southern Italian population, particularly concerning the potential exposure ranges and contextual factors that may confer either risk or protective effects. Given this framework, the present study was carried out to examine the dose–response association between the intake of red meat and MASLD at the survey level in a Southern Italian population, while controlling for a comprehensive set of selected confounding variables. Furthermore, associations with specific categories of red meat were also assessed.

## 2. Materials and Methods

### 2.1. Study Population and Study Design

The NUTRIHEP study is a population-based survey conducted in the municipality of Putignano, Italy, involving adults aged 18 years and older (range: 18–96). Medical records from local general practitioners were extracted to construct the study sample, leveraging Italy’s mandatory family physician system to align medical records with census data and thereby minimize errors in the age and gender distribution of participants. Trained physicians and/or nutritionists administered structured interviews to collect data on sociodemographic characteristics, health status, personal medical history, and lifestyle factors—including smoking status, education level (classified according to the International Standard Classification of Education), marital status, and dietary habits [21].

The study comprised two waves: a baseline assessment conducted in 2004–2005, followed by a second wave between 2014 and 2018 that invited all eligible participants from the first wave to re-enroll. Participants in the second wave underwent the same evaluation protocol as at baseline. Although the study included longitudinal follow-up, the present analysis adopts a cross-sectional design based solely on data from the second wave.

Full methodological details have been published elsewhere [22]. The study was conducted in accordance with the 1975 Declaration of Helsinki, received ethical approval from the Ethics Committee of the National Institute of Gastroenterology and Research Hospital (approvals DDG-CE 502/2005 and DDG-CE-792/2014, issued 20 May 2005 and 14 February 2014, respectively) [22], and obtained written informed consent from all participants after providing complete information about the use of their medical data. A total of 1426 subjects were included in this analysis. Reporting followed the STROBE-nut (Strengthening the Reporting of Observational Studies in Epidemiology—Nutritional Epidemiology) guidelines [23], and participants signed informed consent forms before undergoing examination.

Written informed consent was obtained from all participants following a comprehensive disclosure regarding the use of their medical data for research purposes. A total of 1426 enrolled individuals were included in the present analysis. The study received ethical approval from Ethics Committee of the National Institute of Gastroenterology and Research Hospital (approvals DDG-CE 502/2005 and DDG-CE-792/2014, issued on 20 May 2005 and 14 February 2014) [22]. For the purposes of this analysis, a cross-sectional design was adopted, utilizing data exclusively from the second wave of the study.

### 2.2. Dietary Assessment, Exposure, and Outcome

During the visit, participants completed the validated European Prospective Investigation into Cancer and Nutrition (EPIC) food frequency questionnaire. Responses for all 260 food items were converted into average daily intakes expressed in grams, following a methodology previously applied in other EPIC-based studies. Individual nutrient intakes were then calculated by linking reported foods to the standardized EPIC Nutrient Database [22].

Red meat intake was computed by summing the intake (g/day) of the relative food group items including stewed red meat, roasted red meat, red meat cutlet, red meat slices, rare-cooked red meat steak, medium-cooked red meat steak, well-done red meat steak, red meat hamburger, red meat meatballs, boiled red meat, animal fat, fatty meat, sheep meat, horse meat, liver, giblets, meat sauce on pasta, meat sauce on rice and meat broth.

Notably, we defined red meat as beef, veal, pork, lamb, goat, and horse on the basis of the definition of red meat provided by previous studies [11].

MASLD was diagnosed in participants exhibiting hepatic steatosis on ultrasound in the absence of AFL (alcoholic fatty liver; defined as ≥30 g/day for men or ≥20 g/day for women) [22,24], drug-induced steatosis (e.g., from corticosteroids, valproic acid, or amiodarone), chronic hepatitis B or C infection, or other identifiable causes of liver fat accumulation [25].

### 2.3. Potential Confounders

#### 2.3.1. Food Groups and Single Item Foods

Food groups were formed by summarizing the daily intake of single foods. Concerning that, a number of composite indicators were created [26], such as legumes, vegetables, dairy foods, white meat, processed meat, fish and seafood, fruits, fried foods, grains, soft drinks, and sugar-sweetened foods. All food groups with relative items are presented in Table S1. In addition to food groups, single-item foods such as eggs (g/day), margarine (g/day), and alcohol (g/day) were also considered in the set of potential confounders.

#### 2.3.2. Other Potential Confounders

Beyond dietary factors, the following variables were considered potential confounders: age, sex, education (high school diploma: yes/no), body mass index (BMI), smoking habit (0 = no, 1 = yes), diabetes, total cholesterol levels, and daily energy intake (kcal/day).

### 2.4. Statistical Analysis

Participants’ characteristics were summarized as mean ± standard deviation (SD) for continuous variables and as frequency with percentage (%) for categorical variables. Descriptive statistics were also computed across quartiles of red meat consumption (g/day), both overall and stratified by sex, to preliminarily assess dose–response trends.

Initial dose–response modeling for MASLD (coded 0 = no, 1 = yes) was performed using red meat intake categorized into quartiles, with analyses conducted overall and stratified by sex. Both unadjusted and confounder-adjusted logistic regression models were fitted. The confounder set was selected using a directed acyclic graph (DAG)-based causal inference approach [27,28], which identified a minimally sufficient adjustment set through theory-driven criteria. Potential confounders were initially identified from the literature on comparable study settings [29].

To characterize the shape and magnitude of the association between red meat intake (g/day) and MASLD, adjusted dose–response models for binary outcomes were fitted [30,31]. Missing data were handled via complete case analysis (listwise deletion). Red meat intake was modeled as a continuous variable using restricted cubic splines with nine knots placed at the 1st, 12.5th, 25th, 37.5th, 50th, 62.5th, 75th, 87.5th, and 99th percentiles [29,32], with the median intake serving as the reference value. Odds ratios (ORs) with 95% confidence intervals (CIs) were plotted continuously against red meat consumption; statistical significance was inferred when the 95% CI excluded OR = 1. Covariates in the models were fixed at their median (continuous variables), mode (ordinal variables), or reference category (dichotomous variables) [33]. Notably, the statistical methodology on the application of the restricted cubic spline has also been reported by the epidemiological study by Guido et al. (2024) [34] on the dose–response relationship between white meat intake and MASLD.

In addition, to make the model more parsimonious, we have implemented an AIC model selection on the restricted cubic splines by using (i) four, (ii) five, and (iii) nine knots. In this way, we have verified the robustness of the statistical estimation by the modeling, by following Burnham and Anderson (2004) [35], suggesting the choice of the models with a lower AIC, but also stating the strong support to less parsimonious models when the AIC differences were less than 4.

Finally, as a sensitivity analysis, generalized additive models (GAMs) with a binomial distribution were also fitted to confirm results, reporting ORs as the effect measure.

Prior to modeling, associations between each confounder and red meat intake were evaluated using Pearson/Spearman correlation coefficients, mean differences (MD), or ORs, as appropriate. Multicollinearity was assessed via variance inflation factors (VIFs); variables with VIF > 5 were excluded from final models.

Interaction effects were examined by testing sex (1 = male), age (years), and BMI (kg/m^2^) as potential effect modifiers [36]. For continuous moderators, plots displayed MASLD ORs across red meat intake levels at varying moderator values. For sex (a dichotomous moderator), the plot was inverted to show the sex-specific effect across red meat intake levels; this graphical inversion did not alter the interpretation of the interaction effect, as the statistical model defines the interaction term as the product of the main effects.

An additional in-depth analysis explored the interplay between red meat and grain consumption (g/day), used as a proxy for carbohydrate intake. First, participants were stratified by red meat quartiles, and grain intake was summarized (mean ± SD) and compared across strata using one-way ANOVA. Second, interaction dose–response models for MASLD were fitted with grain intake as a moderator, both in the full sample and stratified by sex [26].

Statistical significance was set at *p* < 0.05; *p*-values between 0.05 and 0.10 were also reported as suggestive of a trend, as suggested by Burdette et al. [37]. All analyses were conducted in R software (version 4.3.3) [38] using the packages dagitty [36], rms [33], mgcv [32], and interactionRCS [39].

## 3. Results

Missing data were handled using listwise deletion, resulting in a sample of 1192 participants (509 males, 42.7%). Among these, 587 individuals (49.2%) had MASLD, comprising 278 males (54.6% of males) and 309 females (45.2% of females). Table 1 presents descriptive statistics for the exposure variables—total red meat intake and its individual components—along with MASLD status and potential confounders, reported as mean ± standard deviation (SD) or frequencies (%), as appropriate. It is worth pointing out that Table 1 reports a number of descriptive statistics already shown in a previous article relative to a study performed on the Nutrihep data [34]. Notably, all individual red meat items exhibited large SDs and coefficients of variation exceeding one, reflecting zero-inflated distributions with substantial overdispersion.

It shows some significant differences in the consumption of some kinds of red meat between men and women (Table 2). In particular, men overall eat more meat sauces on pasta, stewed red meat, roasted red meat, medium-cooked red meat steaks, animal fat, horse meat, liver and giblets, while women seem to prefer well-done steaks, red meatballs, and sheep meat. These findings confirm that the preference for specific foods may be influenced by sex. In Table S2, the pairwise association between exposure and confounders is shown.

**Figure 1:**
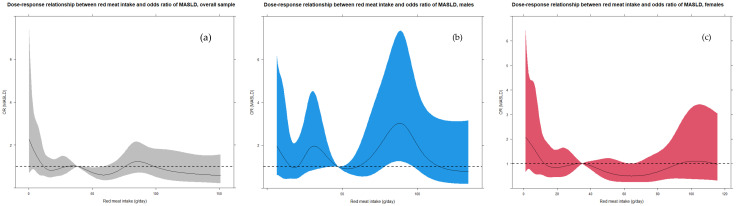
Overall sample and sex-stratified dose–response curves of the relationship between red meat intake and odds ratio of MASLD. (**a**) Dose–response relationship between red meat intake and odds ratio of MASLD, adjusted logistic regression; (**b**) dose–response relationship between red meat intake and odds ratio of MASLD, male, adjusted logistic regression; (**c**) dose–response relationship between red meat intake and odds ratio of MASLD, females, adjusted logistic regression. Black indicated dose–response effect, whereas the 95% confidence bounds are presented in shadowed gray, cyan, and red, respectively. ORs were significant according to whether their 95% confidence bounds did not involve the value “1” (no-association value, dotted black line). OR: odds ratio. MASLD: Non-Alcoholic Fatty Liver Disease. The models are adjusted for potential confounders included in the DAG-related minimal sufficient adjustment set.

Concerning the associations between red meat intake and MASLD, no significant trends (*p* > 0.05) were detected both overall and by sex (see Table 3). Notably, just the model fitted on the overall sample returned a suggestive (0.05 < *p* < 0.10) protective effect in the Q3 (i.e., 50th–75th percentiles class) (OR = 0.686, *p* = 0.078) in relation to the reference class, i.e., 0th–25th percentiles class (Q1). However, all the ORs were mainly and slightly less than one; just in males, the linear effect was slightly positive, close to one (1.002), and the Q2 and Q4 classes (vs Q1) reported ORs equal to 1.204 and 1.482, respectively. In detail, Figure 1 shows that the overall dose–response effect (in cyan) of the “red meat intake—MASLD” relationship is swinging around OR = 1, that is, absence of effect. Analogously, the trend was approximately similar by sex. To be noted, a significant negative effect (OR > 1) emerged by considering intakes between 75 and 90 g/day in males. Remarkably, the significance of the dose–response ORs was evaluated according to whether their 95% confidence bounds did not involve the value “1”, by considering the median of the red meat intake as a reference value. Finally, additional multiple logistic regression models were fitted by dichotomizing the exposure in relation to an intake equal to 50 g/day [11], but no significant results were discovered both in overall sample (OR = 0.885; *p* = 0.612) and stratifying by sex: OR = 1.036 (*p* = 0.883) in males, OR = 0.807 (*p* = 0.556) in females.

Concerning single food items (see Table 3), the intake of boiled red meat has shown a significant negative association with MASLD in the overall sample (OR = 0.711, *p* = 0.047) by considering the dichotomized item at the 75th percentile. At the same time, in females, red meatball intake had a protective effect, both linearly (OR = 0.945, *p* = 0.020) and by considering the 75th percentile-dichotomized item (OR = 0.584, *p* = 0.023). Also, roasted red meat intake presented a suspicious effect by an OR equal to 2.152 (*p* = 0.097), whereas red cutlet intake provided a suggestive protective effect (OR = 0.540, *p* = 0.087), in relation to the dichotomizations at 95th and 90th percentiles, respectively. Finally, the red meat slices intake showed a suggestive negative association with MASLD (OR = 0.952 < 1, *p* = 0.076). In males, liver intake showed a linear protective effect (OR = 0.936, *p* = 0.087).

It is worth pointing out that for the categorization of single food items in percentiles, because of the sparsity, a threshold was applied only in relation to the highest first non-zero percentile by jointly considering overall and sex-stratified samples.

Due to data sparsity in individual food items, percentile-based categorization was implemented using a threshold defined at the highest first non-zero percentile. This threshold was determined by jointly evaluating both the overall sample and sex-stratified subsamples to ensure adequate representation across groups.

The shaded area represents the confidence bands (i.e., confidence intervals for each OR value). For the statistical significance, we judged the OR as significant according to whether its 95% CI included the value “1”. It is worth pointing out that a large 95% CI indicated a small sample size for corresponding values of red meat intake. As for the sex-stratified trends, an approximately decreasing trend was discovered in males, with small- scale fluctuations, whereas an increasing trend was observed in females. In confirmation of this, the first plot in Figure S1 shows that the sex effect (OR, 1 = male) decreases in relation to the intake, and it is significantly bigger than one between 5 and 30 g/day.

Regarding the interaction with age and BMI in the overall sample, no significant effects were revealed, although the spline function had variability. However, after sex stratification, the estimated dose–response relationships provided a more regular shape, as shown in Figure S2.

Finally, in-depth analysis linking red meat consumption with “grains”; Table S3 shows the statistics for each food across quartile-related red meat intake strata. Notably, as red meat intake increased by quartiles, the mean values of “grains” also increased. Figure S3 displays the estimated dose–response relationships between red meat intake and MASLD, both overall and stratified by sex, with grain consumption (g/day) included as a moderator. Notably, among males, red meat intake showed a modest protective association (OR ≈ 0.98) at grain intakes between 100 and 175 g/day, whereas a slight detrimental effect (OR ≈ 1.02) emerged at grain intakes between 210 and 320 g/day.

Regarding spline specification, when model convergence failed, the number of knots in the cubic spline function was reduced by removing knots placed at extreme percentiles where the sample size was sparse. Consequently, interaction dose–response models incorporated fewer knots to ensure stable estimation.

With respect to confounder selection, the DAG-derived minimally sufficient adjustment set for estimating the direct effect of red meat intake on MASLD included the following variables: sex, age, education, BMI, smoking status, diabetes, total cholesterol, processed meat, white meat, alcohol, soft drinks, daily energy intake (kcal), fruits, vegetables, legumes, grains, dairy products, sugar-sweetened foods, fried foods, fish, eggs, and margarine. The R/dagitty code used to generate this DAG is provided in Appendix A. During statistical modeling, daily energy intake was excluded from the adjustment set due to multicollinearity, as it exhibited a variance inflation factor (VIF) > 5.

## 4. Discussion

The present study was conducted with a population of 1192 subjects from the NUTRIHEP sample, which consists of a cohort from a small municipality in southern Italy. The participants had an average age of 55 years and were predominantly overweight. This study aimed to investigate the potential dose–response relationship between the consumption of red meat, specifically unprocessed red meat, and the prevalence of MASLD.

Unexpectedly, the study did not find a statistically significant relationship between the consumption of red meat and MASLD, either in the whole population or when men and women were analyzed separately. In men, the linear model suggested a minimal increase in risk, and some intake categories showed higher odds compared to the lowest intake group, although without a consistent pattern. The dose–response analyses confirmed that there was no clear trend, both overall and when separated by sex. However, in men, a statistically significant increase in MASLD risk was observed at higher intake levels (approximately 75–90 g/day), suggesting a potential negative effect limited to high consumption.

To date, evidence indicates a positive association between red meat and the risk of chronic liver disease, including MASLD [19,40]. However, the association between unprocessed red meat and health outcomes is typically weaker compared to processed red meat. This may be attributed to the higher levels of SFAs and additional non-meat ingredients that are usually added [19,20]. Furthermore, regarding dose–response relationships, Zhou et al. in their recent meta-analysis found no clear linear relationship for unprocessed red meat, which aligns with the overall findings of this study [20].

It is worth noting that the median intake of red meat among participants in the Nutrihep cohort was 44 g per day (approximately 308 g per week), that is, a median intake of 51 g for males and 40 g for females. These intakes align with the recommendations for the general population of 350–500 g per week [11,40,41]. Our results indicate a higher likelihood of MASLD in males who consume greater amounts of red meat. One possible explanation is that a high intake of red meat may increase hepatic iron stores, promoting oxidative stress, a well-established cause of liver damage [41]. Generally, men tend to have higher levels of serum ferritin, which is also associated with a higher risk of metabolic syndrome, overweight, and diabetes [42,43]. Moreover, the android pattern of fat distribution is characterized by a greater accumulation of visceral adipose tissue (VAT), which is accompanied by fat deposition in ectopic sites, including the liver [44]. In contrast, several studies have demonstrated that a gynoid pattern of fat distribution, characterized by a high expandability of subcutaneous adipose tissue (SAT) with preferential fat deposition in the gluteofemoral regions, is associated with a lower risk of cardiometabolic dysfunction when compared with the android pattern. This protective profile has been attributed to the capacity of SAT to buffer excess lipid storage, thereby limiting visceral and ectopic fat accumulation [45].

According to the evidence derived from this study, preferences for different types and methods of preparing red meat appear to vary according to sex. Men generally consume more meat-based pasta sauces, stewed red meat, roasted red meat, medium-cooked steaks, animal fat, horse meat, liver, and offal, whereas women tend to prefer well-done steaks, meatballs made from red meat, and lamb.

These findings support the notion that preferences for specific foods may be influenced by sex. In support of these observations, research over recent decades has highlighted significant sex-related differences in hormonal pathways, medical parameters, dietary preferences, and eating behaviors [46]. Sexual dimorphism in adiposity between men and women appears to be a critical determinant of dietary preferences. Sex hormones play a crucial role in regulating the accumulation and distribution of body fat, which in turn influences food choices. This regulation occurs through the expression of estrogen, progesterone, and androgen receptors in adipose tissue depots [47].

Nonetheless, the analysis of gender differences in food preferences, particularly regarding red meat, is a complex phenomenon that cannot be reduced to a simple biological dichotomy. This type of investigation requires the integration of multiple dimensions and measures of sex, including structural components, such as cultural norms and social gender expectations; social factors, such as relational contexts and social roles; and individual aspects, which encompass cognitive processes, personal motivations, and acquired behaviors [48].

Another factor that can contribute to these results is provided by a recent investigation into sex-related differences in eating behaviors, according to which men tend to report a greater perception of hunger during the late afternoon and in the pre-dinner period, whereas women exhibit a higher perception of hunger in the morning hours [47]. From a chrononutritional perspective, this temporal pattern of food intake is clinically relevant, as consuming meals later in the day has been associated with adverse metabolic outcomes. Late meal consumption has been linked to increased abdominal adiposity and a higher prevalence of metabolic disorders related to metabolic syndrome, as demonstrated in the analyses of NHANES 2015–2018 data [49].

In any case, the data that have emerged are open to further investigation in the vast and partially unexplored field of gender research.

The confounders examined in this study have been extensively researched as potential factors in the development of MASLD related to red meat intake. Blood TG levels are particularly relevant, as MASLD develops from TG accumulation in hepatocytes. Free fatty acids derived from the diet, adipose tissue, and de novo lipogenesis are converted to TG in the liver, leading to MASLD. Evidence suggests that red meat intake can elevate blood TG levels: a recent meta-analysis of randomized controlled trials reported higher TG with red meat consumption [50], and a cross-sectional study of middle-aged men found that those with the highest red meat and saturated fat intake had a greater prevalence of hypertriglyceridaemia [51].

High-density lipoprotein cholesterol (HDL-C) is a traditional lipid marker with good predictive value for MASLD, allowing early identification of high-risk individuals [52]. Regarding red meat, a 13-year prospective cohort in Iran showed that higher consumption was associated with modest increases in HDL-C and other lipid parameters [53], while a metabolomics study found red meat intake linked to variations in multiple lipoprotein markers, including HDL subtypes [54].

T2DM is another key confounder. Both processed and unprocessed red meat consumption is associated with increased risk of T2DM [55], which frequently coexists with MASLD. At the same time, T2DM and MASLD share mechanisms such as insulin resistance, chronic low-grade inflammation, and altered lipid metabolism [56].

BMI also plays a dual role in the association between red meat consumption and MASLD. First of all, a greater BMI, specifically greater than or equal to 25, is one of the metabolic factors included in the diagnostic criteria for MASLD [9]. Furthermore, weight loss has been shown to significantly reduce hepatic steatosis [25]. Secondly, red meat intake has been linked to higher BMI, waist circumference, and risk of overweight [57,58], probably because it is often associated with an unhealthier dietary pattern, greater total caloric intake, less whole grains, less plant-based proteins, less fruits, and less vegetables [59].

Moreover, unhealthy behaviors such as alcohol consumption and smoking might also mediate the results of this study. Alcohol consumption above certain thresholds is used to distinguish between MASLD and Alcoholic Liver Disease (ALD) and is a key dietary factor in the management of the disease itself. Additionally, alcohol intake and smoking often correlate with higher red meat consumption and lower vegetable intake [60]. Finally, educational attainment is also another important confounder: higher education is strongly protective against MASLD, likely by influencing healthier dietary choices, including reduced red meat consumption [61].

Analysis of the results suggests that certain cuts and methods of preparing red meat may be associated with a potentially higher risk of MASLD, assuming the influence of variables such as the origin of the animal, the cut of meat, the method of preparation, such as boiling, grilling, frying [62,63], or the addition of other ingredients in recipes, such as breadcrumbs, cheese, eggs, or oils, on the overall metabolic effects. However, it was not possible to investigate these hypotheses in depth in this study.

Finally, in the study, cereal consumption was considered a moderating variable to evaluate whether the association between red meat intake and the risk of MASLD varied with different levels of cereal consumption. The moderating variable “cereals” includes foods such as bread, pasta, and other cereal-based products and, from a nutritional perspective, represents the dietary intake of carbohydrates. In this regard, results among males indicated that at moderate cereal consumption levels, specifically between 100 g/day and 175 g/day, the association between red meat intake and MASLD was slightly protective. In contrast, at higher levels of cereal consumption, that is, between 210 g/day and 320 g/day, the observed effect tended to become moderately adverse. These findings can be interpreted in light of established evidence highlighting the influence of meal composition on postprandial metabolic responses [64]. In particular, balanced meals containing moderate proportions of protein, lipids, and carbohydrates, when compared to meals that are predominantly rich in carbohydrates, are linked to a reduction in postprandial glycemic response (PPGR), more efficient control of the glycemic index and glycemic load of the meal, and favorable modulation of insulin secretion [65]. These mechanisms may contribute to more stable metabolic responses and mitigate risk factors associated with hepatic and metabolic dysfunction.

Ultimately, this research may provide further evidence to assess the potential impact of red meat consumption on the development of MASLD in a population such as the Nutrihep cohort, which is characterized at baseline by a high adherence to the Mediterranean Diet, which already represents the key nutritional strategy in the non-pharmacological management of MASLD [66]. Emphasizing a dietary approach that shifts the traditional Mediterranean pattern further towards a ‘green’ Mediterranean Diet, while discouraging high consumption of red meat, both processed and unprocessed, may help prevent the development of MASLD, as already demonstrated in previous studies [67].

### Strengths and Limitations

This study presents several notable strengths but also inevitable limitations. A key strength lies in the large sample size, coupled with an important geographical factor: the study population is drawn from a town in the Apulian region. From an anthropological perspective, this setting likely demonstrates a strong adherence to the lifestyle habits characteristic of the Mediterranean pattern. This has allowed us to define a population in terms of eating habits across age groups.

In addition, red meat intake was assessed using the validated European Prospective Investigation into Cancer and Nutrition (EPIC) food frequency questionnaire, an instrument adopted in the large European cohort studies coordinated by the International Agency for Research on Cancer (IARC) of the World Health Organization. This questionnaire represents a key tool for investigating the relationship between dietary patterns and epidemiologically relevant health outcomes.

From a methodological perspective, a DAG was employed to identify potential confounders while minimizing distortion. This causal inference approach substantially enhances the robustness and credibility of the study’s findings. In addition, an in-depth analysis was conducted to examine the interaction between the consumption of red meat and the “cereals” (i.e., grains) food group, which served as a moderator in the relationship between red meat intake and MASLD.

Next, due to data sparsity in individual food items, percentile-based categorization was implemented using a threshold defined at the highest first non-zero percentile. We are aware that this data-driven approach may introduce bias, but for this study, in our opinion, it is the best trade-off to provide preliminary and explorative evidence.

However, the study is observational and cross-sectional; therefore, it is not possible to establish a causal relationship between red meat intake and the risk of MASLD. Furthermore, dietary data were self-reported, which introduces a potential for bias, although the data collection phase was reviewed by registered dietitian nutritionists. Finally, the absence of physical activity data constitutes an additional limitation, given its known influence on MASLD outcomes.

Finally, it is worth pointing out that the study did not establish a consistent trend between red meat consumption and the incidence of MASLD in the overall study population or when analyzed by sex. The findings support the idea that dietary preferences might be influenced by sex.

## 5. Conclusions

This study revealed a suspicious effect of red meat consumption on MASLD in men, specifically at intakes ranging from 75 to 90 g/day. Nevertheless, this research did not establish a consistent trend between red meat consumption and the incidence of MASLD in the overall study population or when analyzed by sex.

Our findings indicate a significant gender-related differentiation in red meat consumption preferences, including both the specific types favored and the preparation methods employed. These findings support the idea that dietary preferences may be influenced by sex.

This study contributes to the scientific debate by highlighting the need for additional observational and interventional research to better investigate the relationship between red meat and MASLD, elucidate potential causal mechanisms, and enhance our understanding of the interaction between sex and red meat consumption in relation to MASLD. It also underscores the importance of examining sex-related differences in preferences for red meat products from organic, sociocultural, and anthropological viewpoints.

## Figures and Tables

**Table 1:** Preliminary statistics.

Variables	Overall Sample (*n* = 1192)	Males (*n* = 509, 42.7%)	Females (*n* = 683, 57.3%)	*p*-Value * (Males vs. Females)
	Mean ± SD	Mean ± SD	Mean ± SD	
**Exposure**				
*Red meat intake (g/day)*	*44.86 ± 31.23*	*51.39 ± 34.22*	*39.99 ± 27.85*	**<0.001**
Meat sauce on pasta (g/day)	2.531 ± 5.119	3.616 ± 6.536	1.722 ± 3.522	**<0.001**
Meat sauce on rice (g/day)	1.617 ± 2.638	1.709 ± 3.161	1.549 ± 2.168	0.327
Meat broth (g/day)	2.699 ± 7.135	3.114 ± 8.063	2.39 ± 6.345	*0.094*
Stewed red meat (g/day)	1.913 ± 4.197	2.341 ± 4.799	1.593 ± 3.656	**0.003**
Roasted red meat (g/day)	4.556 ± 7.041	5.523 ± 8.248	3.836 ± 5.889	<0.001
Boiled red meat (g/day)	2.045 ± 4.885	2.348 ± 5.796	1.819 ± 4.067	*0.078*
Red meat cutlet (g/day)	1.687 ± 3.031	1.936 ± 3.634	1.501 ± 2.475	**0.020**
Red meat slices (g/day)	2.606 ± 5.463	3.176 ± 6.896	2.182 ± 4.034	**0.003**
Rare-cooked red meat steak (g/day)	0.751 ± 3.667	1.238 ± 4.008	0.389 ± 3.347	**<0.001**
Medium-cooked red meat steak (g/day)	2.695 ± 5.351	3.428 ± 6.18	2.149 ± 4.568	**<0.001**
Well-done red meat steak (g/day)	1.661 ± 4.468	1.3 ± 4.018	1.93 ± 4.761	**0.013**
Red meat hamburger (g/day)	1.494 ± 2.898	1.319 ± 2.58	1.625 ± 3.11	*0.064*
Red meat meatballs (g/day)	3.004 ± 4.510	2.566 ± 3.939	3.331 ± 4.87	**0.003**
Animal fat (g/day)	0.568 ± 0.894	0.754 ± 1.075	0.429 ± 0.699	**<0.001**
Fatty meat (g/day)	6.762 ± 7.811	7.521 ± 7.803	6.196 ± 7.775	**0.003**
Sheep meat (g/day)	2.263 ± 3.986	2.805 ± 4.415	1.859 ± 3.585	**<0.001**
Horse meat (g/day)	4.312 ± 8.53	4.635 ± 8.968	4.072 ± 8.186	0.266
Liver (g/day)	1.239 ± 3.157	1.504 ± 3.567	1.042 ± 2.799	**0.015**
Giblets (g/day)	0.456 ± 1.22	0.562 ± 1.276	0.377 ± 1.172	**0.011**
**Outcome**	Mean ± SD or *n* (%)	Mean ± SD or *n* (%)	Mean ± SD or *n* (%)	
MASLD (yes/no) (%)	587/605 (49.2/50.7)	278/231 (54.6/45.4)	309/374 (45.2/54.8)	**0.001**
**Confounders**				
Age (y)	54.65 ± 14.37	55.1 ± 15.19	54.32 ± 13.738	0.361
Education (high school or higher: yes/no) (%)	570/622 (47.8/52.2)	254/255 (49.9/50.1)	316/367 (46.3/53.7)	0.236
Body Mass Index (kg/m^2^)	27.61 ± 4.981	27.93 ± 4.413	27.37 ± 5.357	*0.050*
Smoking (yes/no) (%)	146/1046 (12.2/87.8)	81/428 (15.9/84.1)	65/618 (9.5/90.5)	**<0.001**
Diabetes (yes/no) (%)	80/1112 (6.7/93.3)	37/472 (7.3/92.7)	43/640 (6.3/93.7)	0.506
Cholesterol (mg/dL)	191.4 ± 36.061	187.5 ± 37.095	194.4 ± 35.012	**0.001**
Daily caloric intake (kcal/day)	2045 ± 737.13	2207 ± 780.52	1924.2 ± 678.87	**<0.001**
Alcohol (g/day)	10.41 ± 19.96	18.499 ± 27.28	4.38 ± 7.522	**<0.001**
Dairy foods (g/day)	118.33 ± 105.51	112.2 ± 101.16	122.9 ± 108.48	*0.078*
Fish (g/day)	37.91 ± 25.59	38.37 ± 26.74	37.57 ± 24.71	0.598
Fruits (g/day)	297 ± 169.19	311 ± 187.6	286.5 ± 153.4	**0.016**
Fried foods (g/day)	6.45 ± 8.76	7.38 ± 8.71	5.758 ± 8.73	**0.001**
Eggs (g/day)	19.77 ± 15.34	19.37 ± 18.37	20.06 ± 12.62	0.468
Grains (g/day)	183.6 ± 97.98	203.7 ± 108.02	168.7 ± 86.89	**<0.001**
Legumes (g/day)	38.58 ± 31.36	42.57 ± 36.15	35.6 ± 26.9	<0.001
Margarine (g/day)	0.048 ± 0.339	0.05 ± 0.352	0.046 ± 0.330	0.871
Soft drinks (g/day)	67.8 ± 144.36	70.23 ± 160.15	65.98 ± 131.46	0.625
Sugar-sweetened foods (g/day)	82.23 ± 67.13	84.36 ± 71.85	80.64 ± 63.38	0.353
Vegetables (g/day)	205.5 ± 111.47	193.5 ± 106.21	214.4 ± 14.48	**0.001**
*White meat intake (g/day)*	*34.07 ± 26.33*	*35.81 ± 28.68*	*32.77 ± 24.37*	*0.053*
Stewed white meat (g/day)	0.925 ± 2.445	1.068 ± 2.841	0.819 ± 2.099	*0.095*
Roasted white meat (g/day)	2.407 ± 5.534	2.399 ± 4.68	2.412 ± 6.096	0.968
White meat cutlet (g/day)	0.808 ± 1.803	0.804 ± 1.793	0.811 ± 1.811	0.946
White meat slices (g/day)	1.42 ± 4.176	1.255 ± 3.022	1.542 ± 4.859	0.209
Rare-cooked white meat steak (g/day)	0.362 ± 2.18	0.654 ± 2.958	0.144 ± 1.293	**<0.001**
Medium-cooked white meat steak (g/day)	1.363 ± 3.48	1.663 ± 3.945	1.139 ± 3.072	**0.013**
Well-done white meat steak (g/day)	0.847 ± 2.534	0.565 ± 1.935	1.057 ± 2.884	**<0.001**
White meat hamburger (g/day)	0.744 ± 1.772	0.562 ± 1.399	0.879 ± 1.996	**0.001**
White meat meatballs (g/day)	1.331 ± 2.513	1.066 ± 2.161	1.528 ± 2.732	**0.001**
Chicken thigh (g/day)	5.983 ± 13.98	6.551 ± 15.929	5.559 ± 12.341	0.243
Chicken breast (g/day)	4.748 ± 11.464	4.508 ± 11.596	4.926 ± 11.371	0.535
Chicken—other parts (g/day)	2.175 ± 7.367	1.621 ± 6.747	2.587 ± 7.776	**0.022**
Poultry (g/day)	7.612 ± 14.797	9.294 ± 16.958	6.358 ± 12.824	**0.001**
Chicken skin (g/day)	0.418 ± 0.681	0.5684 ± 0.776	0.305 ± 0.576	**<0.001**
Rabbit (g/day)	2.925 ± 4.456	3.23 ± 4.713	2.698 ± 4.243	**0.044**
*Processed meat intake (g/day)*	*28.4 ± 27.87*	*33.16 ± 29.92*	*24.86 ± 25.7*	**<0.001**
Cotechino or zampone (g/day)	8.37 ± 8.863	9.186 ± 9.689	7.761 ± 8.148	**0.007**
Canned meat (g/day)	0.494 ± 2.069	0.597 ± 2.285	0.418 ± 1.889	0.152
Cured meat sandwich (g/day)	6.953 ± 13.581	9.288 ± 15.691	5.213 ± 11.471	**<0.001**
Ham (g/day)	2.825 ± 4.339	2.735 ± 4.025	2.892 ± 4.561	0.530
Lean ham (g/day)	3.385 ± 5.376	4.065 ± 6.499	2.878 ± 4.29	**<0.001**
Cured meat sausages (g/day)	1.438 ± 3.148	1.602 ± 2.638	1.317 ± 3.47	0.107
Mortadella (g/day)	1.289 ± 2.857	1.597 ± 3.021	1.06 ± 2.708	**0.001**
Bresaola (g/day)	1.854 ± 3.405	1.823 ± 3.535	1.876 ± 3.307	0.792
Soppressata (g/day)	0.932 ± 2.185	1.251 ± 2.641	0.695 ± 1.735	**<0.001**
Other cured meat (g/day)	0.639 ± 2.424	0.734 ± 2.993	0.568 ± 1.890	0.273
Fatty ham (g/day)	0.221 ± 0.369	0.277 ± 0.397	0.178 ± 0.34	**<0.001**

**Note.** SD: standard deviation, *n*: sample size, y: years, MASLD: metabolic dysfunction-associated steatotic liver disease. **Bold** highlights significant results (*p* < 0.05), and *italics* indicate suggestive results (0.05 < *p* < 0.10). * indicates two-tailed unpaired *t*-test or chi-square test, as appropriate.

**Table 2:** Statistics for quartiles of red meat intake, for the overall sample and by sex.

	Overall Sample					Males					Females					Males vs. Females
	Min	1st Q	2nd Q	3rd Q	Max	Min	1st Q	2nd Q	3rd Q	Max	Min	1st Q	2nd Q	3rd Q	Max	*p*-Value *
**Red meat (g/day)**	**0**	**23.1**	**38.3**	**59.8**	**350**	**0**	**26.6**	**47.2**	**67**	**350**	**0**	**21.35**	**35**	**52.55**	**267.7**	**<0.001**
Meat sauce on pasta (g/day)	0	0	0.7	2.9	74	0	0	1.2	4.4	74	0	0	0.4	1.9	28.9	**<0.001**
Meat sauce on rice (g/day)	0	0.1	0.7	2	39.3	0	0.1	0.7	1.9	39.3	0	0.2	0.7	2	14.5	0.643
Meat broth (g/day)	0	0	0	2.5	106.5	0	0	0	3.1	106.5	0	0	0	2.05	70	0.251
Stewed red meat (g/day)	0	0	0	2.2	52.6	0	0	0	2.7	52.6	0	0	0	1.8	37.1	**0.036**
Roasted red meat (g/day)	0	0	2.1	5.7	64.3	0	0	2.7	7.1	64.3	0	0	1.8	5.05	57.1	**<0.001**
Boiled red meat (g/day)	0	0	0	2.6	78.9	0	0	0	2.8	78.9	0	0	0	2.3	50.8	0.384
Red meat cutlet (g/day)	0	0	0.6	2.1	38.5	0	0	0.6	2.4	38.5	0	0	0.5	2.1	17.1	0.241
Red meat slices (g/day)	0	0	0.7	2.9	68.6	0	0	0.9	3.2	68.6	0	0	0.6	2.4	32.7	*0.071*
Rare-cooked red meat steak (g/day)	0	0	0	0	73.1	0	0	0	0	30.5	0	0	0	0	73.1	**<0.001**
Medium-cooked red meat steak (g/day)	0	0	0	3.1	45.7	0	0	0	4.8	45.7	0	0	0	2.1	34.3	**<0.001**
Well-done red meat steak (g/day)	0	0	0	1.425	54.5	0	0	0	0	45	0	0	0	2	54.5	**<0.001**
Red meat hamburger (g/day)	0	0	0	1.8	27.2	0	0	0	1.6	21.4	0	0	0.1	1.8	27.2	*0.082*
Red meat meatballs (g/day)	0	0	1.6	3.925	54	0	0	1.3	3.5	29.9	0	0	1.8	4.3	54	**0.001**
Animal fat (g/day)	0	0	0.2	0.8	11.9	0	0	0.4	1.1	11.9	0	0	0.1	0.6	5.6	**<0.001**
Fatty meat (g/day)	0	1.1	3.3	10	57.1	0	2.7	6.7	10	42.9	0	0.5	3.3	10	57.1	**<0.001**
Sheep meat (g/day)	0	0	0.7	2.1	43.3	0	0.4	1.1	4.3	37.1	0	0	0.7	1.8	43.3	**<0.001**
Horse meat (g/day)	0	0	0.8	4.8	82.9	0	0	1.2	4.8	82.9	0	0	0.8	4.8	62.1	**0.003**
Liver (g/day)	0	0	0	1	51.4	0	0	0.3	1.3	51.4	0	0	0	0.7	34.3	**<0.001**
Giblets (g/day)	0	0	0	0.5	14.3	0	0	0	0.5	14.3	0	0	0	0.3	13.3	**<0.001**

**Note**. **Bold** highlights significant results (*p* < 0.05), and *italics* mark suggestive results (0.05 < *p* < 0.10). Min: minimum. Max: maximum. Q: quartile. * indicates two-tailed Mann–Whitney test.

**Table 3:** Results of logistic regression models on MASLD: raw, adjusted, and quartiles categorization effects.

	Overall Sample (*n* = 1197, 587 MASLD)			Males (*n* = 509, 278 MASLD)			Females (*n* = 653, 309 MASLD)		
Food Item	Raw Models	Adjusted Models	Quartiles Categorized EXPOSURE *	Raw Models	Adjusted Models	Quartiles Categorized Exposure *	Raw Models	Adjusted Models	Quartiles Categorized Exposure *
	OR *p*-Value 95% CI	OR *p*-Value 95% CI	OR * p-Value 95% CI	OR *p*-Value 95% CI	OR *p*-Value 95% CI	OR * p-Value 95% CI	OR *p*-Value 95% CI	OR *p*-Value 95% CI	OR * p-Value 95% CI
Red meat (g/day)	0.998 0.404 0.994; 1.002	0.996 0.262 0.991; 1.002	OR_25–50_ = 0.990 0.962 0.659; 1.487 *OR_50–75_ = 0.686* *0.078* *0.451; 1.044* OR_75–100_ = 0.879 0.580 0.558; 1.386	0.997 0.256 0.992; 1.002	1.002 0.654 0.993; 1.009	OR_25–50_ = 1.204 0.565 0.638; 2.271 OR_50–75_ = 0.869 0.675 0.452; 1.672 OR_75–100_ = 1.482 0.255 0.752; 2.9217	0.998 0.373 0.992; 1.003	0.994 0.184 0.986; 1.003	OR_25–50_ = 0.653 0.163 0.359; 1.189 OR_50–75_ = 0.601 0.166 0.293; 1.234 OR_75–100_ = 0.568 0.327 0.184; 1.757
Meat sauce on pasta (g/day)	1.004 0.661 0.982; 1.027	0.990 0.510 0.961; 1.019	^ OR_50–100_ = 0.875 0.382 0.647; 1.181	1.002 0.881 0.975; 1.029	1.001 0.977 0.966; 1.035	^ OR_50–100_ = 0.779 0.289 0.4917; 1.236	0.988 0.601 0.947; 1.032	0.964 0.254 0.906; 1.026	^ OR_75–100_ = 0.721 0.191 0.441; 1.178
Meat sauce on rice (g/day)	*0.957* *0.066* *0.915*; *1.003*	0.960 0.177 0.905; 1.018	^ OR_50–100_ = 1.040 0.791 0.776; 1.395	0.964 0.222 0.911; 1.022	0.985 0.710 0.9113; 1.065	^ OR_50–100_ = 1.274 0.294 0.809; 2.005	0.943 0.112 0.878; 1.014	0.928 0.129 0.843; 1.022	^ OR_50–100_ = 0.897 0.595 0.602; 1.337
Meat broth (g/day)	1.004 0.629 0.988; 1.020	0.992 0.473 0.972; 1.013	^ OR_75–100_ = 0.840 0.307 0.601; 1.173	1.002 0.851 0.980; 1.024	0.988 0.430 0.958; 1.018	^ OR_75–100_ = 0.707 0.199 0.417; 1.201	1.003 0.782 0.979; 1.027	1.004 0.800 0.973; 1.036	^ OR_75–100_ = 0.813 0.359 0.522; 1.265
Stewed red meat (g/day)	1.006 0.630 0.979; 1.034	0.983 0.359 0.948; 1.019	^ OR_75–100_ = 0.814 0.240 0.578; 1.147	0.997 0.903 0.962; 1.034	0.994 0.828 0.943; 1.047	^ OR_75–100_ = 0.824 0.477 0.484; 1.403	1.009 0.658 0.968; 1.052	0.976 0.433 0.919; 1.037	^ OR_75–100_ = 0.856 0.504 0.542; 1.351
Roasted red meat (g/day)	1.003 0.646 0.987; 1.020	1.017 0.110 0.996; 1.039	^ OR_50–100_ = 0.972 0.849 0.726; 1.301	1.004 0.689 0.983; 1.026	1.024 0.112 0.994; 1.054	^ OR_50–100_ = 1.139 0.571 0.725; 1.791	0.995 0.720 0.970; 1.021	1.013 0.976 0.978; 1.049	*^ OR_95–100_ = 2.152* *0.097* *0.871*; *5.321*
Boiled red meat (g/day)	0.981 0.137 0.956; 1.006	0.971 0.131 0.934; 1.008	**^ OR_75–100_ = 0.711** **0.047** **0.508; 0.995**	*0.969* *0.090* *0.934*; *1.004*	0.974 0.375 0.921; 1.031	^ OR_75–100_ = 0.664 0.122 0.396; 1.116	0.991 0.632 0.954; 1.029	0.965 0.223 0.912; 1.022	^ OR_75–100_ = 0.884 0.595 0.562; 1.3918939594
Red meat cutlet (g/day)	0.971 0.149 0.935; 1.010	0.978 0.381 0.933; 1.026	^ OR_50–100_ = 0.891 0.433 0.666; 1.190	0.973 0.274 0.926; 1.021	0.986 0.658 0.928; 1.048	^ OR_50–100_ = 0.937 0.779 0.596; 1.474	0.958 0.186 0.900; 1.021	0.966 0.437 0.885; 1.054	*^ OR_90–100_ = 0.540* *0.087* *0.267*; *1.092*
Red meat slices (g/day)	**0.953** **<0.001** **0.929; 0.978**	*0.974* *0.091* *0.946*; *1.004*	*^ OR_50–100_ = 0.747* *0.053* *0.556*; *1.004*	**0.958** **0.006** **0.929; 0.987**	0.981 0.296 0.948; 1.016	^ OR_50–100_ = 0.822 0.407 0.518; 1.305	**0.935** **0.002** **0.896; 0.977**	*0.952* *0.076* *0.901*; *1.005*	^ OR_75–100_ = 0.706 0.161 0.434; 1.150
Rare-cooked red meat steak (g/day)	1.009 0.565 0.977; 1.041	1.011 0.564 0.974; 1.049	^ OR_95–100_ = 1.528 0.216 0.780; 2.992	1.006 0.784 0.962; 1.051	1.010 0.755 0.947; 1.076	^ OR_90–100_ = 1.156 0.712 0.534; 2.505	1.001 0.979 0.957; 1.047	1.007 0.768 0.959; 1.058	^ OR_95–100_ = 1.170 0.720 0.496; 2.762
Medium-cooked red meat steak (g/day)	0.997 0.820 0.976; 1.018	0.998 0.892 0.971; 1.026	^ OR_75–100_ = 0.854 0.356 0.612; 1.193	0.991 0.540 0.963; 1.019	0.994 0.766 0.958; 1.032	^ OR_75–100_ = 1.006 0.980 0.594; 1.707	0.996 0.821 0.963; 1.030	1.014 0.525 0.970; 1.061	^ OR_75–100_ = 1.189 0.448 0.760; 1.860
Well-done red meat steak (g/day)	0.995 0.750 0.971; 1.021	0.994 0.727 0.961; 1.028	^ OR_75–100_ = 0.803 0.194 0.576; 1.118	0.966 0.146 0.922; 1.012	0.985 0.614 0.929; 1.043	^ OR_80–100_ = 0.978 0.940 0.562; 1.703	1.015 0.334 0.984; 1.049	1.002 0.942 0.959; 1.046	^ OR_80–100_ = 0.729 0.200 0.449; 1.182
Red meat hamburger (g/day)	*0.966* *0.099* *0.928*; *1.006*	1.019 0.425 0.971; 1.070	^ OR_75–100_ = 1.069 0.700 0.759; 1.506	0.972 0.412 0.908; 1.040	1.021 0.616 0.938; 1.112	^ OR_75–100_ = 0.992 0.976 0.589; 1.669	0.968 0.211 0.921; 1.018	1.021 0.514 0.959; 1.085	^ OR_95–100_ = 1.403 0.426 0.609; 3.232
Red meat meatballs (g/day)	0.992 0.528 0.967; 1.017	0.981 0.262 0.948; 1.014	^ OR_50–100_ = 0.863 0.322 0.645; 1.154	1.031 0.187 0.984; 1.080	1.048 0.134 0.985; 1.115	^ OR_50–100_ = 1.167 0.500 0.743; 1.832	0.978 0.173 0.946; 1.010	**0.945** **0.020** **0.902; 0.992**	**^ OR_75–100_ = 0.584** **0.023** **0.367; 0.928**
Animal fat (g/day)	1.008 0.899 0.888; 1.144	0.926 0.431 0.767; 1.119	^ OR_75–100_ = 0.802 0.151 0.593; 1.084	0.936 0.430 0.795; 1.102	1.026 0.844 0.788; 1.337	^ OR_50–100_ = 0.939 0.790 0.591; 1.492	1.032 0.771 0.832; 1.280	0.781 0.133 0.567; 1.078	^ OR_75–100_ = 0.774 0.324 0.465; 1.288
Fatty meat (g/day)	1.009 0.189 0.995; 1.024	1.000 0.983 0.980; 1.020	^ OR_50–100_ = 0.881 0.407 0.654; 1.187	0.999 0.998 0.977; 1.022	1.013 0.389 0.983; 1.044	^ OR_50–100_ = 0.982 0.943 0.596; 1.617	1.013 0.164 0.994; 1.033	0.992 0.597 0.964; 1.021	^ OR_50–100_ = 0.765 0.195 0.511; 1.147
Sheep meat (g/day)	**1.045** **0.005** **1.013; 1.078**	1.014 0.421 0.979; 1.051	^ OR_50–100_ = 1.113 0.478 0.827; 1.498	**1.049** **0.038** **1.003; 1.098**	1.031 0.274 0.975; 1.089	^ OR_50–100_ = 1.047 0.842 0.661; 1.659	1.031 0.159 0.987; 1.077	0.998 0.940 0.948; 1.051	^ OR_50–100_ = 0.991 0.964 0.664; 1.478
Horse meat (g/day)	0.991 0.209 0.977; 1.004	0.994 0.584 0.975; 1.014	^ OR_50–100_ = 0.950 0.737 0.706; 1.280	0.998 0.824 0.978; 1.017	1.019 0.228 0.988; 1.049	^ OR_50–100_ = 1.234 0.371 0.779; 1.957	*0.984* *0.097* *0.964*; *1.003*	0.976 0.109 0.948; 1.005	^ OR_50–100_ = 0.905 0.636 0.601; 1.365
Liver (g/day)	0.983 0.388 0.948; 1.020	0.980 0.428 0.932; 1.030	^ OR_75–100_ = 1.105 0.571 0.781; 1.566	*0.945* *0.065* *0.891*; *1.004*	*0.936* *0.087* *0.867*; *1.009*	^ OR_50–100_ = 0.741 0.196 0.471; 1.167	1.014 0.621 0.961; 1.069	1.029 0.405 0.961; 1.101	^ OR_75–100_ = 1.316 0.243 0.829; 2.089
Giblets (g/day)	1.017 0.720 0.926; 1.116	0.964 0.569 0.851; 1.093	^ OR_75–100_ = 0.997 0.989 0.676; 1.472	1.011 0.870 0.882; 1.161	0.971 0.768 0.799; 1.180	^ OR_75–100_ = 1.045 0.874 0.608; 1.792	1.001 0.992 0.880; 1.138	0.922 0.380 0.769; 1.105	^ OR_75–100_ = 1.034 0.894 0.633; 1.689

**Note**. **Bold** highlights significant results (*p* < 0.05), *italics* mark suggestive ones (0.05 < *p* < 0.10). 95% CI: 95% confidence interval. * The models are adjusted for the potential confounders included in the DAG-related minimal sufficient adjustment set. ^ Zero-inflated predictor: categorized only on the median or 3rd quartile, by using the lower category as reference. If this categorization returned non-different ‘breaks’, percentiles were applied (e.g., 95%). Lower-case numbers are relative to the upper class of the food item after dichotomization by percentile-based threshold.

## Data Availability

The original contributions presented in this study are included in the article. Further inquiries can be directed to the corresponding author.

## Supplementary Materials

The following supporting information can be downloaded at: https://www.mdpi.com/article/10.3390/nu18061002/s1. Figure S1: Interaction effects of sex, age and BMI with red meat intake on MASLD; Figure S2: Interaction effects of age and BMI with red meat intake on MASLD by sex; Figure S3: Interaction effects of grains with red meat intake on MASLD, overall and by sex; Table S1: Food groups confounders; Table S2: Association between exposure and confounders; Table S3: Subject grain (carbohydrates) profiling by daily red meat intake quartiles stratification.

## Appendix A. R/Dagitty Code on the Direct Acyclic Graph (DAG) for Theory-Driven Minimal Sufficient Adjustment Set of the Confounders of the Direct Effect of the Red Meat Intake on MASLD

dag { “Dairy products” [adjusted, pos = “−2.101, −0.269”] “Fresh fruits” [adjusted, pos = “0.062, 1.458”] “Fried foods” [adjusted, pos = “−2.132, −1.270”] “Kcal intake” [adjusted, pos = “0.327, 1.163”] “Processed meat” [adjusted, pos = “0.635, −0.368”] “Red meat” [exposure, pos = “0.528, 0.291”] “Soft drinks” [adjusted, pos = “−2.106, 0.159”] “Sugar foods” [adjusted, pos = “0.135, −1.882”] “White meat” [adjusted, pos = “0.546, −0.869”] Age [adjusted, pos = “−2.264, 0.739”] Alcool [adjusted, pos = “−1.607, −1.765”] BMI [adjusted, pos = “0.582, 1.076”] Cholesterol [adjusted, pos = “−1.522, 1.324”] Diabetes [adjusted, pos = “−1.915, 1.280”] Education [adjusted, pos = “−1.719, −1.284”] Eggs [adjusted, pos = “−1.239, −1.962”] Fish [adjusted, pos = “−0.288, −1.937”] Grains [adjusted, pos = “−2.041, −0.836”] Legumes [adjusted, pos = “0.558, −1.696”] Margarin [adjusted, pos = “−1.075, 1.227”] Sex [adjusted, pos = “−0.330, −1.713”] Smoke [adjusted, pos = “−0.824, −1.706”] MASLD [outcome, pos = “−0.400, −0.315”] Vegetables [adjusted, pos = “−0.173, 0.779”] “Dairy products” → MASLD “Dairy products” ↔ “Fresh fruits” “Dairy products” ↔ “Red meat” “Dairy products” ↔ “Sugar foods” “Dairy products” ↔ “White meat” “Dairy products” ↔ Eggs “Dairy products” ↔ Fish “Dairy products” ↔ Legumes “Dairy products” ↔ Vegetables “Fresh fruits” ↔ “Processed meat” “Fresh fruits” ↔ “Red meat” “Fresh fruits” ↔ Eggs “Fresh fruits” ↔ Fish “Fresh fruits” ↔ Grains “Fresh fruits” ↔ Legumes “Fresh fruits” ↔ Vegetables “Fried foods” ↔ “Red meat” “Fried foods” ↔ “White meat” “Fried foods” ↔ Eggs “Fried foods” ↔ Fish “Fried foods” ↔ Margarin “Kcal intake” → MASLD “Kcal intake” ↔ “Processed meat” “Kcal intake” ↔ “Red meat” “Kcal intake” ↔ “White meat” “Processed meat” → MASLD “Processed meat” ↔ “Sugar foods” “Processed meat” ↔ Cholesterol “Processed meat” ↔ Vegetables “Red meat” → “Processed meat” “Red meat” → MASLD “Red meat” ↔ “Sugar foods” “Red meat” ↔ Cholesterol “Red meat” ↔ Eggs “Red meat” ↔ Fish “Red meat” ↔ Grains “Red meat” ↔ Legumes “Red meat” ↔ Margarin “Red meat” ↔ Vegetables “Soft drinks” → MASLD “Soft drinks” ↔ “Sugar foods” “Soft drinks” ↔ Age “Soft drinks” ↔ Diabetes “Sugar foods” → MASLD “Sugar foods” ↔ “White meat” “Sugar foods” ↔ Diabetes “Sugar foods” ↔ Eggs “Sugar foods” ↔ Fish “Sugar foods” ↔ Grains “White meat” → “Processed meat” “White meat” → MASLD “White meat” ↔ Eggs “White meat” ↔ Fish “White meat” ↔ Grains “White meat” ↔ Legumes Age → MASLD Alcool → MASLD Alcool ↔ Education Alcool ↔ Smoke BMI → MASLD Diabetes → MASLD Education → MASLD Education ↔ Sex Eggs ↔ Fish Eggs ↔ Grains Eggs ↔ Legumes Eggs ↔ Vegetables Fish ↔ Grains Fish ↔ Legumes Fish ↔ Vegetables Grains → MASLD Grains ↔ Margarin Grains ↔ Vegetables Legumes ↔ Margarin Legumes ↔ Vegetables Margarin → MASLD Margarin ↔ Vegetables Sex → MASLD Smoke → MASLD }

## References

[1] Rinella M.E., Lazarus J.V., Ratziu V., Francque S.M., Sanyal A.J., Kanwal F., Romero D., Abdelmalek M.F., Anstee Q.M., Arab J.P. (2023). A Multisociety Delphi Consensus Statement on New Fatty Liver Disease Nomenclature. Hepatology.

[2] Dietrich P., Hellerbrand C. (2014). Non-Alcoholic Fatty Liver Disease, Obesity and the Metabolic Syndrome. Best Pract. Res. Clin. Gastroenterol..

[3] Xiao J., Wang F., Yuan Y., Gao J., Xiao L., Yan C., Guo F., Zhong J., Che Z., Li W. (2025). Epidemiology of Liver Diseases: Global Disease Burden and Forecasted Research Trends. Sci. China Life Sci..

[4] Zhang H., Zhou X.-D., Shapiro M.D., Lip G.Y.H., Tilg H., Valenti L., Somers V.K., Byrne C.D., Targher G., Yang W. (2024). Global Burden of Metabolic Diseases, 1990–2021. Metabolism.

[5] Vos M.B., Abrams S.H., Barlow S.E., Caprio S., Daniels S.R., Kohli R., Mouzaki M., Sathya P., Schwimmer J.B., Sundaram S.S. (2017). NASPGHAN Clinical Practice Guideline for the Diagnosis and Treatment of Nonalcoholic Fatty Liver Disease in Children: Recommendations from the Expert Committee on NAFLD (ECON) and the North American Society of Pediatric Gastroenterology, Hepatology and Nutrition (NASPGHAN). J. Pediatr. Gastroenterol. Nutr..

[6] Berná G., Romero-Gomez M. (2020). The Role of Nutrition in Non-alcoholic Fatty Liver Disease: Pathophysiology and Management. Liver Int..

[7] Powell E.E., Wong V.W.-S., Rinella M. (2021). Non-Alcoholic Fatty Liver Disease. Lancet.

[8] Brunetto M.R., Salvati A., Petralli G., Bonino F. (2023). Nutritional Intervention in the Management of Non-Alcoholic Fatty Liver Disease. Best Pract. Res. Clin. Gastroenterol..

[9] Tacke F., Horn P., Wai-Sun Wong V., Ratziu V., Bugianesi E., Francque S., Zelber-Sagi S., Valenti L., Roden M., Schick F. (2024). EASL–EASD–EASO Clinical Practice Guidelines on the Management of Metabolic Dysfunction-Associated Steatotic Liver Disease (MASLD). J. Hepatol..

[10] Henney A.E., Gillespie C.S., Alam U., Hydes T.J., Cuthbertson D.J. (2023). Ultra-Processed Food Intake Is Associated with Non-Alcoholic Fatty Liver Disease in Adults: A Systematic Review and Meta-Analysis. Nutrients.

[11] Aicr, WCRF World Cancer Research Fund/American Institute for Cancer Research. Diet, Nutrition, Physical Activity and Cancer: A Global Perspective. Continuous Update Project Expert Report 2018. https://www.wcrf.org/research-policy/global-cancer-update-programme/.

[12] Godfray H.C.J., Aveyard P., Garnett T., Hall J.W., Key T.J., Lorimer J., Pierrehumbert R.T., Scarborough P., Springmann M., Jebb S.A. (2018). Meat Consumption, Health, and the Environment. Science.

[13] Liang M., Wu J., Li H., Zhu Q. (2024). *N*-glycolylneuraminic Acid in Red Meat and Processed Meat Is a Health Concern: A Review on the Formation, Health Risk, and Reduction. Compr. Rev. Food Sci. Food Saf..

[14] Ruiz H.H., Ramasamy R., Schmidt A.M. (2020). Advanced Glycation End Products: Building on the Concept of the “Common Soil” in Metabolic Disease. Endocrinology.

[15] Vijay A., Al-Awadi A., Chalmers J., Balakumaran L., Grove J.I., Valdes A.M., Taylor M.A., Shenoy K.T., Aithal G.P. (2022). Development of Food Group Tree-Based Analysis and Its Association with Non-Alcoholic Fatty Liver Disease (NAFLD) and Co-Morbidities in a South Indian Population: A Large Case-Control Study. Nutrients.

[16] Hashemian M., Merat S., Poustchi H., Jafari E., Radmard A.-R., Kamangar F., Freedman N., Hekmatdoost A., Sheikh M., Boffetta P. (2021). Red Meat Consumption and Risk of Nonalcoholic Fatty Liver Disease in a Population with Low Meat Consumption: The Golestan Cohort Study. Am. J. Gastroenterol..

[17] Guo X., Yin X., Liu Z., Wang J. (2022). Non-Alcoholic Fatty Liver Disease (NAFLD) Pathogenesis and Natural Products for Prevention and Treatment. Int. J. Mol. Sci..

[18] Alawadi A.A., Vijay A., Grove J.I., Taylor M.A., Aithal G.P. (2025). The Development of a Food-Group, Tree Classification Method and Its Use in Exploring Dietary Associations with Metabolic Dysfunction-Associated Steatotic Liver Disease (MASLD) and Other Health-Related Outcomes in a UK Population. Metabol. Open.

[19] Ivancovsky-Wajcman D., Fliss-Isakov N., Grinshpan L.S., Salomone F., Lazarus J.V., Webb M., Shibolet O., Kariv R., Zelber-Sagi S. (2022). High Meat Consumption Is Prospectively Associated with the Risk of Non-Alcoholic Fatty Liver Disease and Presumed Significant Fibrosis. Nutrients.

[20] Zhou Q., Hu H., Hu L., Liu S., Chen J., Tong S. (2024). Association between Processed and Unprocessed Red Meat Consumption and Risk of Nonalcoholic Fatty Liver Disease: A Systematic Review and Dose-Response Meta-Analysis. J. Glob. Health.

[21] Cozzolongo R., Osella A.R., Elba S., Petruzzi J., Buongiorno G., Giannuzzi V., Leone G., Bonfiglio C., Lanzilotta E., Manghisi O.G. (2009). Epidemiology of HCV Infection in the General Population: A Survey in a Southern Italian Town. Am. J. Gastroenterol..

[22] Donghia R., Campanella A., Bonfiglio C., Cuccaro F., Tatoli R., Giannelli G. (2024). Protective Role of Lycopene in Subjects with Liver Disease: NUTRIHEP Study. Nutrients.

[23] Lachat C., Hawwash D., Ocké M.C., Berg C., Forsum E., Hörnell A., Larsson C.l., Sonestedt E., Wirfält E., Åkesson A. (2016). Strengthening the Reporting of Observational Studies in Epidemiology—Nutritional Epidemiology (STROBE-nut): An Extension of the STROBE Statement. Nutr. Bull..

[24] Younossi Z., Anstee Q.M., Marietti M., Hardy T., Henry L., Eslam M., George J., Bugianesi E. (2018). Global Burden of NAFLD and NASH: Trends, Predictions, Risk Factors and Prevention. Nat. Rev. Gastroenterol. Hepatol..

[25] Chalasani N., Younossi Z., Lavine J.E., Charlton M., Cusi K., Rinella M., Harrison S.A., Brunt E.M., Sanyal A.J. (2018). The Diagnosis and Management of Nonalcoholic Fatty Liver Disease: Practice Guidance from the American Association for the Study of Liver Diseases. Hepatology.

[26] Zupo R., Sardone R., Donghia R., Castellana F., Lampignano L., Bortone I., Misciagna G., De Pergola G., Panza F., Lozupone M. (2020). Traditional Dietary Patterns and Risk of Mortality in a Longitudinal Cohort of the Salus in Apulia Study. Nutrients.

[27] Perković E., Textor J., Kalisch M., Maathuis M.H. (2015). A Complete Generalized Adjustment Criterion. arXiv.

[28] Pearl J. (2009). Causality.

[29] Donghia R., Pesole P.L., Coletta S., Bonfiglio C., De Pergola G., De Nucci S., Rinaldi R., Giannelli G. (2023). Food Network Analysis in Non-Obese Patients with or without Steatosis. Nutrients.

[30] Desquilbet L., Mariotti F. (2010). Dose-response Analyses Using Restricted Cubic Spline Functions in Public Health Research. Stat. Med..

[31] Ruppert D., Wand M.P., Frontmatter R.J.C. (2003). Semiparametric Regression.

[32] Wood S.N. (2017). Generalized Additive Models: An Introduction with R.

[33] Harrell F.E. (2024). Rms: Regression Modeling Strategies. R Package Version 6.8-0. https://cran.r-project.org/package=rms.

[34] Guido D., Cerabino N., Di Chito M., Donghia R., Randazzo C., Bonfiglio C., Giannelli G., De Pergola G. (2024). A Dose–Response Study on the Relationship between White Meat Intake and Metabolic Dysfunction-Associated Steatotic Liver Disease (MASLD) in Southern Italy: Results from the Nutrihep Study. Nutrients.

[35] Burnham K.P., Anderson D.R. (2004). Multimodel Inference: Understanding AIC and BIC in Model Selection. Sociol. Methods Res..

[36] Textor J., van der Zander B., Gilthorpe M.S., Liśkiewicz M., Ellison G.T.H. (2016). Robust Causal Inference Using Directed Acyclic Graphs: The R Package ‘Dagitty’. Int. J. Epidemiol..

[37] Burdette W.J., Gehan E.A. (1970). Planning and Analysis of Clinical Studies.

[38] R Core Team (2020). R: A Language and Environment for Statistical Computing.

[39] Melloni G., Bellavia A., Xiong H. (2023). InteractionRCS: Calculate Estimates in Models with Interaction. R Package Version 0.1.1. https://cran.r-project.org/web/packages/interactionRCS/index.html.

[40] Nyamsuren U., Peng Y., Shin S. (2025). Association Between Meat Intake and Metabolic Dysfunction-Associated Steatotic Liver Disease Incidence in a Korean Population From the Health Examinees Study. Mol. Nutr. Food Res..

[41] Gensluckner S., Wernly B., Datz C., Aigner E. (2024). Iron, Oxidative Stress, and Metabolic Dysfunction—Associated Steatotic Liver Disease. Antioxidants.

[42] Iglesias-Vázquez L., Arija V., Aranda N., Aglago E.K., Cross A.J., Schulze M.B., Quintana Pacheco D., Kühn T., Weiderpass E., Tumino R. (2022). Factors Associated with Serum Ferritin Levels and Iron Excess: Results from the EPIC-EurGast Study. Eur. J. Nutr..

[43] Han L., Wang Y., Li J., Zhang X., Bian C., Wang H., Du S., Suo L. (2014). Gender Differences in Associations of Serum Ferritin and Diabetes, Metabolic Syndrome, and Obesity in the China Health and Nutrition Survey. Mol. Nutr. Food Res..

[44] Gastaldelli A., Cusi K., Pettiti M., Hardies J., Miyazaki Y., Berria R., Buzzigoli E., Sironi A.M., Cersosimo E., Ferrannini E. (2007). Relationship Between Hepatic/Visceral Fat and Hepatic Insulin Resistance in Nondiabetic and Type 2 Diabetic Subjects. Gastroenterology.

[45] Abraham T.M., Pedley A., Massaro J.M., Hoffmann U., Fox C.S. (2015). Association Between Visceral and Subcutaneous Adipose Depots and Incident Cardiovascular Disease Risk Factors. Circulation.

[46] Szadvári I., Ostatníková D., Babková Durdiaková J. (2023). Sex Differences Matter: Males and Females Are Equal but Not the Same. Physiol. Behav..

[47] Lombardo M., Feraco A., Armani A., Camajani E., Gorini S., Strollo R., Padua E., Caprio M., Bellia A. (2024). Gender Differences in Body Composition, Dietary Patterns, and Physical Activity: Insights from a Cross-Sectional Study. Front. Nutr..

[48] Feraco A., Gorini S., Camajani E., Filardi T., Karav S., Cava E., Strollo R., Padua E., Caprio M., Armani A. (2024). Gender Differences in Dietary Patterns and Physical Activity: An Insight with Principal Component Analysis (PCA). J. Transl. Med..

[49] Bernardes da Cunha N., Teixeira G.P., Madalena Rinaldi A.E., Azeredo C.M., Crispim C.A. (2023). Late Meal Intake Is Associated with Abdominal Obesity and Metabolic Disorders Related to Metabolic Syndrome: A Chrononutrition Approach Using Data from NHANES 2015–2018. Clin. Nutr..

[50] Sun L., Yuan J.-L., Chen Q.-C., Xiao W.-K., Ma G.-P., Liang J.-H., Chen X.-K., Wang S., Zhou X.-X., Wu H. (2022). Red Meat Consumption and Risk for Dyslipidaemia and Inflammation: A Systematic Review and Meta-Analysis. Front. Cardiovasc. Med..

[51] Cocate P.G., Natali A.J., de Oliveira A., Alfenas R.d.C.G., Peluzio M.d.C.G., Longo G.Z., dos Santos E.C., Buthers J.M., de Oliveira L.L., Hermsdorff H.H.M. (2015). Red but Not White Meat Consumption Is Associated with Metabolic Syndrome, Insulin Resistance and Lipid Peroxidation in Brazilian Middle-Aged Men. Eur. J. Prev. Cardiol..

[52] Lu S., Xie Q., Kuang M., Hu C., Li X., Yang H., Sheng G., Xie G., Zou Y. (2023). Lipid Metabolism, BMI and the Risk of Nonalcoholic Fatty Liver Disease in the General Population: Evidence from a Mediation Analysis. J. Transl. Med..

[53] Hassannejad R., Moosavian S.P., Mohammadifard N., Mansourian M., Roohafza H., Sadeghi M., Sarrafzadegan N. (2021). Long-Term Association of Red Meat Consumption and Lipid Profile: A 13-Year Prospective Population-Based Cohort Study. Nutrition.

[54] Pan L., Chen L., Lv J., Pang Y., Guo Y., Pei P., Du H., Yang L., Millwood I.Y., Walters R.G. (2022). Association of Red Meat Consumption, Metabolic Markers, and Risk of Cardiovascular Diseases. Front. Nutr..

[55] Shi W., Huang X., Schooling C.M., Zhao J.V. (2023). Red Meat Consumption, Cardiovascular Diseases, and Diabetes: A Systematic Review and Meta-Analysis. Eur. Heart J..

[56] Jing M., Jiang Y. (2025). Microbiome−mediated Crosstalk between T2DM and MASLD: A Translational Review Focused on Function. Front. Endocrinol..

[57] Rouhani M.H., Salehi-Abargouei A., Surkan P.J., Azadbakht L. (2014). Is There a Relationship between Red or Processed Meat Intake and Obesity? A Systematic Review and Meta-analysis of Observational Studies. Obes. Rev..

[58] Wang Z., Zhang B., Wang H., Zhang J., Du W., Su C., Zhang J., Zhai F. (2013). Study on the Multilevel and Longitudinal Association between Red Meat Consumption and Changes in Body Mass Index, Body Weight and Risk of Incident Overweight among Chinese Adults. Zhonghua Liu Xing Bing Xue Za Zhi.

[59] Kim M.N., Lo C.-H., Corey K.E., Luo X., Long L., Zhang X., Chan A.T., Simon T.G. (2022). Red Meat Consumption, Obesity, and the Risk of Nonalcoholic Fatty Liver Disease among Women: Evidence from Mediation Analysis. Clin. Nutr..

[60] Grosso G., Micek A., Godos J., Pajak A., Sciacca S., Galvano F., Boffetta P. (2017). Health Risk Factors Associated with Meat, Fruit and Vegetable Consumption in Cohort Studies: A Comprehensive Meta-Analysis. PLoS ONE.

[61] Donghia R., Bonfiglio C., Giannelli G., Tatoli R. (2025). Impact of Education on Metabolic Dysfunction-Associated Steatotic Liver Disease (MASLD): A Southern Italy Cohort-Based Study. J. Clin. Med..

[62] Liu G., Zong G., Hu F.B., Willett W.C., Eisenberg D.M., Sun Q. (2017). Cooking Methods for Red Meats and Risk of Type 2 Diabetes: A Prospective Study of U.S. Women. Diabetes Care.

[63] Momal U., Naeem H., Aslam F., Shahbaz M., Imran M., Hussain M., Ahmad A., Memon A.G., Mujtaba A., Atif M. (2025). Recent Perspectives on Meat Consumption and Cancer Proliferation. J. Food Process. Preserv..

[64] Wolever T.M., Zurbau A., Koecher K., Au-Yeung F. (2024). The Effect of Adding Protein to a Carbohydrate Meal on Postprandial Glucose and Insulin Responses: A Systematic Review and Meta-Analysis of Acute Controlled Feeding Trials. J. Nutr..

[65] Kdekian A., Alssema M., Van Der Beek E.M., Greyling A., Vermeer M.A., Mela D.J., Trautwein E.A. (2020). Impact of Isocaloric Exchanges of Carbohydrate for Fat on Postprandial Glucose, Insulin, Triglycerides, and Free Fatty Acid Responses—A Systematic Review and Meta-Analysis. Eur. J. Clin. Nutr..

[66] Arita V.A., Cabezas M.C., Hernández Vargas J.A., Trujillo-Cáceres S.J., Mendez Pernicone N., Bridge L.A., Raeisi-Dehkordi H., Dietvorst C.A.W., Dekker R., Uriza-Pinzón J.P. (2025). Effects of Mediterranean Diet, Exercise, and Their Combination on Body Composition and Liver Outcomes in Metabolic Dysfunction-Associated Steatotic Liver Disease: A Systematic Review and Meta-Analysis of Randomized Controlled Trials. BMC Med..

[67] Yaskolka Meir A., Rinott E., Tsaban G., Zelicha H., Kaplan A., Rosen P., Shelef I., Youngster I., Shalev A., Blüher M. (2021). Effect of Green-Mediterranean Diet on Intrahepatic Fat: The DIRECT PLUS Randomised Controlled Trial. Gut.

