# Supplementary material for: A Dose–Response Study on the Relationship Between Red Meat Intake and Metabolic Dysfunction-Associated Steatotic Liver Disease (MASLD) in Southern Italy: Results from the Nutrihep Study
Source: Nutrients. 2026 Mar 21;18(6):1002. doi: 10.3390/nu18061002 (PMC13028667; doi:10.3390/nu18061002)
Supplement: Supplementary file 1 [file nutrients-18-01002-s001.zip › nutrients-4155222-supplementary.pdf]

Supplementary Materials

Table S1. Food groups confounders.

Grains	Vegetables	Legumes	Dairy products	Fried foods	Fish	Eggs	Fruits	Soft drinks	Sugar foods	Margarine
Pastasciutta	Tomato pasta	Pasta legumes	White butter	Fish bastones	Shrimp	Eggs	Apple	Orange squash	Honey	White margarine
Egg pasta	Vegetables pasta	Legumes rice	Tomato butter	Fryed potatoes	Octopes-seps	Mayo	Pear	Fruit juices	Sugar milk	Margarine tomato
Filled pasta	Vegetables rice	Minestra of legumes	Butter ragu	Potato croquettes	Mussels-clams	Omelette	Banana	Soft drinks	Sweetened coffee	Margarina ragu
Rice	Minestron	Dried beans	Butter vegetables		Preserved fish		Kiwi		Sugar yogurt	Vegetable margarine legums
Rice salad	Vegetable broth	Fresh beans fried	Butter minestron		Boxed fish		Oranges grapefruit		Sugar cafelatte	Margarine minestron
Pasta in soup	Tomatoes in canned	Canned beans	Added butter		Sogliola fish		Mandarins		Sugar tea/infusions	Wet margarine
Polenta	Tomatoes end stage	Dried faves	Grated cheese		Sardines		Grapes		Squeezed sugar	Fried margarine
Pizza cut	Salad	Fried fresh favors	Fontina fontal		Trout		Peaches		Biscuits	
Pizza from pizzeria	Raw Pepper	Tinned Broad Beans	Pecorino Caciotta		Salmon		Apricots		Brioches	
Homemade pizza	Raw onions	Dried lentils	Emmenthal groviera		Spadish		Plums		Marmalade	
Chips or salads	Crucible carrofs	Fresh fried lentils	Caciocaval		Other types of fish		Strawberries		Nutella	
Common breads	Crucible fennel	Lentils in squash	Parmigiano				Melon		Filled cake	

White bread	Crudied sedans	Dried chick-peas	Other types of matured cheese				Fruit salad		Unstuffed cake	
Whole-grain sandwiches	Soybeans	Frozen chick-peas	Sottilets						Spoon pie	
Whole grain crackers	Crucible cabbage	Canned chick-peas	Philadelfia						Dry pastries	
Whole bread	Crude carrots	Dried peas	Crescence						Filled cookies	
Conditioned breads	Onion or leek sauces	Fresh frozen peas	Mozzarella						Chocolate	
Toasts	Cooked carrots	Canned peas	Taleggio						Caramels	
Corn flakes	Broccoli		Gorgonzola						Summer ice cream	
Muesli oatmeal fritters	Brussels sprouts		Robiola						Ice cream in winter	
Whole grains	Cauliflower		Ricotta							
Seeds	Rapa seeds		Other types of spruce cheeses							
	Black cabbage		White whole milk							
	Virls		Partially skimmed white milk							
	Spinach		Skimmed white milk							
	Cooked pepper		Whole fruit yogurt							
	Aubergine		Fruit yogurt skimmed							
	Green bean		Yoghurt other whole							
	Zucchini		Yogurt other low-fat							
	Cooked artichokes		Butter							
	Cooked fennel		Fryed butter							
	Red beets		Moist butter							
	Cooked onions		Sandwiches with cheese							

	Vegetable pies									
	Sandwiches with vegetables									

Table S2. Association between exposure and confounders.

1st part	White meat	Processed meat	AGE	Sex (1=male)	Education (1= High School or more)	BMI	Smoke (1=yes)	Diabetes	Cholesterol	Alcohol	Soft Drinks
Red meat	**R=0.348 P<0.001 95%CI=[0.297; 0.397]**	**R=0.324 <0.001 0.272; 0.37**	**R=-0.128 <0.001 -0.184; -0.072**	**MD=11.401 <0.001 7.763; 15.038**	MD=-2.002 0.268 -5.553; 1.549	R=0.030 0.286 0.087; -0.025	**MD=9.985 0.002 3.650; 16.321**	R=-4.066 P=0.267 -11.303; 3.169	R=-0.017 0.551 -0.073; 0.039	**R=0.195 <0.001 0.139; 0.249**	**R=0.084 0.004 0.027; 0.140**
White meat		**R=0.276 <0.001 0.222; 0.327**	**R=-0.278 P <0.001 -0.330; -0.225**	*MD=3.045 P=0.053 -0.049; 6.139*	**MD=3.621 P=0.017 0.638; 6.605**	R=0.045 0.118 -0.011; 0.101	*MD=5.060 P=0.061 -0.234; 10.355*	**MD=-4.739 <0.001 -35.496; -32.503**	R=0.015 0.588 -0.041; 0.072	R=0.047 0.102 -0.009; 0.103	**R=0.096 <0.001 0.040; 0.152**
Processed meat	-	-	**R=-0.329 <0.001 -0.378; -0.277**	**MD=8.298 <0.001 5.058; 11.538**	**MD=8.761 <0.001 5.613; 11.909**	R=-0.019 0.519 -0.075; 0.038	**MD=10.212 <0.001 4.407; 16.016**	**MD=-8.765 0.002 14.202; 3.327**	R=0.016 0.559 -0.039; 0.073	**R=0.144 <0.001 0.088; 0.199**	**R=0.158 <0.001 0.102; 0.213**
AGE	-	-	-	MD=0.779 0.362 -0.896; 2.456	**MD=-13.8 <0.001 -15.241; -12.359**	**R=0.294 <0.001 0.241; 0.345**	**MD=-4.691 <0.001 -7.209; -2.171**	**MD=12.645 <0.001 10.354; 14.936**	**R=0.064 0.025 0.008; 0.121**	**R=0.084 0.003 0.028; 0.141**	**R=-0.141 <0.001 -0.196; -0.084**
Sex	-	-	-	-	X^2= 1.402 0.236	*MD=0.556 0.051 0.000; 1.112*	X^2=**10.516 0.001**	X^2= 0.299 0.584	**MD=-6.863 0.001 -11.023; -2.700**	**MD=14.118 <0.001 11.677; 16.560**	MD=4.251 0.652 -12.821; 21.325
Education	-	-	-	-	-	**-2.852 <0.001 -3.392; -2.312**	**X^2= 5.033 0.024**	**X^2= 18.889 <0.001**	MD=-2.312 0.268 -6.407; 1.782	MD=-1.392 0.237 -3.702; 0.917	MD=7.827 0.351 -8.624; 24.28
BMI	-	-	-	-	-	-	MD=-0.640 0.123	**MD=4.531 <0.001**	**R=0.095 0.001**	R=0.019 0.509	R=0.042 0.143

							-1.456; 0.175	**3.236; 5.825**	**0.038; 0.151**	-0.037; 0.075	-0.014; 0.09
Smoke	-	-	-	-	-	-	-	X²< 0.001<br>1	MD=-2.873<br>0.345<br>-8.871; 3.123	**MD=6.746**<br>**<0.001**<br>**3.355; 10.137**	MD=23.836<br>0.227<br>-15.049; 62.721
Diabetes	-	-	-	-	-	-	-	-	**MD=-17.441**<br>**<0.001**<br>**-25.504; -9.377**	MD=2.073<br>0.305<br>-1.915; 6.061	MD=-17.222<br>0.298<br>-49.937; 15.491
Cholesterol	-	-	-	-	-	-	-	-	-	R=-0.018<br>0.516<br>-0.075; 0.038	R=0.022<br>0.444<br>-0.034; 0.078
Alcohol	-	-	-	-	-	-	-	-	-	-	**R=0.126**<br>**<0.001**<br>**0.070; 0.182**
Soft drink	-	-	-	-	-	-	-	-	-	-	-
Energy intake	-	-	-	-	-	-	-	-	-	-	-
Dairy foods	-	-	-	-	-	-	-	-	-	-	-
Sugar foods	-	-	-	-	-	-	-	-	-	-	-
Fruits	-	-	-	-	-	-	-	-	-	-	-
Vegetables	-	-	-	-	-	-	-	-	-	-	-
Legumes	-	-	-	-	-	-	-	-	-	-	-
Grains	-	-	-	-	-	-	-	-	-	-	-
Fried foods	-	-	-	-	-	-	-	-	-	-	-
Fish	-	-	-	-	-	-	-	-	-	-	-
Eggs	-	-	-	-	-	-	-	-	-	-	-
2nd part											

	**Energy intake**	**Dairy products**	**Sugar foods**	**Fruits**	**Vegetables**	**Legumes**	**Grains**	**Fried foods**	**Fish**	**Eggs**	**Margarine**
Red meat	**R=0.393<br><0.001<br>0.344; 0.440**	R=0.0346<br>0.231<br>-0.022; 0.091	**R=0.105<br><0.001<br>0.048; 0.161**	R=-0.021<br>0.479<br>-0.077; 0.036	**R=0.083<br>0.003<br>0.027; 0.139**	**R=0.134<br><0.001<br>0.078; 0.190**	**R=0.249<br><0.001<br>0.195; 0.301**	**R=0.239<br><0.001<br>0.185; 0.292**	**R=0.130<br><0.001<br>0.074; 0.185**	**R=0.120<br><0.001<br>0.064; 0.176**	R=0.047<br>0.106<br>-0.009; 0.103
White meat	**R=0.312<br><0.001<br>0.260; 0.363**	R=0.010<br>0.705<br>-0.045; 0.067	**R=0.100<br><0.001<br>0.044; 0.156**	R=0.004<br>0.896<br>-0.053; 0.060	**R=0.206<br><0.001<br>0.151; 0.260**	**R=0.135<br><0.001<br>0.077; 0.189**	**R=0.126<br><0.001<br>0.070; 0.182**	**R=0.078<br>0.006<br>0.022; 0.135**	**R=0.259<br><0.001<br>0.206; 0.311**	**R=0.214<br><0.001<br>0.159; 0.267**	R=-0.018<br>0.517<br>-0.075; 0.038
Processed meat	**R=0.464<br><0.001<br>0.418; 0.507**	R=0.034<br>0.235<br>-0.022; 0.090	**R=0.213<br><0.001<br>0.159; 0.267**	*R=-0.050<br>0.078<br>-0.107; 0.006*	**R=0.091<br>0.002<br>0.035; 0.147**	R=0.027<br>0.342<br>-0.029; 0.084	**R=0.289<br><0.001<br>0.236; 0.340**	**R=0.256<br><0.001<br>0.202; 0.308**	**R=0.098<br><0.001<br>0.041; 0.154**	**R=0.121<br><0.001<br>0.064; 0.176**	R=0.015<br>0.611<br>-0.042; 0.071
AGE	**R=-0.181<br><0.001<br>-0.235; -0.125**	**R=0.082<br>0.005<br>0.025; 0.138**	**R=-0.189<br><0.001<br>-0.243; -0.134**	**R=0.201<br><0.001<br>0.146; 0.255**	**R=-0.121<br><0.001<br>-0.176; -0.064**	R=0.022<br>0.439<br>-0.034; 0.079	**R=-0.113<br><0.001<br>-0.169; -0.057**	**R=-0.097<br><0.001<br>-0.152; -0.040**	**R=-0.121<br><0.001<br>-0.176; -0.064**	**R=-0.096<br><0.001<br>-0.152; -0.039**	*R=0.052<br>0.072<br>-0.004; 0.109*
Sex	**MD=283.01<br><0.001<br>198.11; 367.905**	*MD=-10.7711<br>0.078<br>-22.760; 1.218*	MD=3.718<br>0.353<br>-4.137; 11.574	**MD=24.457<br>0.016<br>4.484; 44.429**	**MD=-20.948<br>0.001<br>-33.565; 8.331**	**MD=6.969<br><0.001<br>3.231; 10.707**	**MD=34.988<br><0.001<br>23.548; 46.428**	**MD=1.621<br>0.002<br>0.6193; 2.624**	MD=0.798<br>0.598<br>-2.177; 3.774	MD=-0.687<br>0.468<br>-2.545; 1.171	MD=0.003<br>0.872<br>-0.036; 0.042
Education	**MD=116.989<br>0.006<br>33.370; 200.607**	MD=-7.595<br>0.213<br>-19.546; 4.356	**MD=12.039<br>0.001<br>4.437; 19.640**	**MD=-29.457<br>0.003<br>-48.680; -10.234**	**MD=21.254<br>0.001<br>8.585; 33.923**	MD=-1.037<br>0.566<br>-4.590; 2.515	**MD=12.512<br>0.027<br>1.411; 23.614**	**MD=-29.457<br>0.003<br>-48.680; -10.234**	**MD=4.655<br>0.002<br>1.751; 7.559**	**MD=1.987<br>0.027<br>0.223; 3.751**	MD=-0.027<br>0.159<br>-0.065; 0.010
BMI	R=-0.032<br>0.266<br>-0.088; 0.024	R=0.022<br>0.433<br>-0.034; 0.079	**R=-0.119<br><0.001**	**R=0.067<br>0.019<br>0.011; 0.124**	R=0.022<br>0.439<br>-0.034; 0.079	R=0.047<br>0.101	R=-0.021<br>0.466	R=-0.020<br>0.482<br>-0.077; 0.036	R=0.011<br>0.701<br>-0.045; 0.067	R=-0.021<br>0.464	R=0.030<br>0.287

			**-0.175; -0.063**			-0.009; 0.104	-0.077; 0.035			-0.077; 0.035	-0.026; 0.087
Smoke	**MD=225.018<br>0.002<br>82.176; 367.86**	MD=-5.353<br>0.567<br>- 23.791; 13.085	MD=5.058<br>0.467<br>-8.660; 18.778	*MD=-26.686<br>0.095<br>-58.109; 4.737*	MD=0.021<br>0.998<br>-19.692; 19.735	MD=3.908<br>0.286<br>-3.314; 11.131	**MD=26.158<br>0.011<br>6.058; 46.259**	MD=0.641<br>0.483<br>-1.159; 2.441	MD=0.475<br>0.839<br>-4.143; 5.095	MD=-0.567<br>0.695<br>-3.429; 2.293	MD=0.032<br>0.426<br>-0.047; 0.112
Diabetes	**MD=-189.486<br>0.041<br>-371.087; -7.884**	MD=3.218<br>0.778<br>-19.429; 25.865	**MD=-32.606<br><0.001<br>-45.047; -20.165**	MD=22.840<br>0.223<br>-14.111; 59.792	MD=11.833<br>0.411<br>-16.649; 40.316	MD=0.963<br>0.748<br>- 4.982; 6.910	MD=-12.852<br>0.299<br>-37.343; 11.638	MD=0.038<br>0.968<br>-1.883; 1.960	MD=2.195<br>0.474<br>-3.882; 8.273	*MD=-2.633<br>0.057<br>-5.355; 0.088*	MD=0.097<br>0.171<br>-0.042; 0.236
Cholesterol	R=0.013<br>0.653<br>-0.043; 0.069	R=0.0117<br>0.686<br>-0.045; 0.068	R=-0.004<br>0.885<br>-0.061; 0.052	R=0.039<br>0.174<br>-0.0173; 0.096	R=0.032<br>0.268<br>-0.024; 0.088	R=0.009<br>0.761<br>-0.047; 0.065	R=0.010<br>0.721<br>-0.046; 0.067	R=0.0112<br>0.697<br>-0.045; 0.067	R=-0.002<br>0.949<br>-0.058; 0.054	R=0.014<br>0.624<br>-0.042; 0.070	R=-0.030<br>0.286<br>-0.087; 0.025
Alcohol	**R=0.327<br><0.001<br>0.275; 0.376**	R=0.009<br>0.766<br>-0.048; 0.065	R=0.005<br>0.861<br>-0.051; 0.061	R=0.031<br>0.284<br>-0.025; 0.087	R=-0.018<br>0.522<br>-0.075; 0.038	**R=0.062<br>0.030<br>0.006; 0.119**	**R=0.096<br><0.001<br>0.039; 0.152**	**R=0.086<br>0.003<br>0.030; 0.142**	R=0.043<br>0.132<br>-0.013; 0.100	R=0.027<br>0.337<br>-0.029; 0.08	R=-0.017<br>0.539<br>-0.074; 0.039
Soft drinks	**R=0.284<br><0.001<br>0.232; 0.337**	**R=0.093<br>0.001<br>0.036; 0.149**	**R=0.147<br><0.001<br>0.091; 0.202**	**R=0.069<br>0.017<br>0.012; 0.125**	R=0.045<br>0.115<br>-0.011; 0.102	R=0.045<br>0.123<br>-0.012; 0.101	**R=0.076<br>0.008<br>0.019; 0.132**	**R=0.100<br><0.001<br>0.043; 0.156**	**R=0.063<br>0.029<br>0.006; 0.119**	**R=0.089<br>0.002<br>0.033; 0.145**	R=0.035<br>0.217<br>-0.021; 0.092
Energy intake	-	**R=0.301<br><0.001<br>0.249; 0.352**	**R=0.586<br><0.001<br>0.548; 0.622**	**R=0.294<br><0.001<br>0.242; 0.345**	**R=0.426<br><0.001<br>0.378; 0.471**	**R=0.286<br><0.001<br>0.233; 0.337**	**R=0.688<br><0.001<br>0.657; 0.717**	**R=0.239<br><0.001<br>0.184; 0.291**	**R=0.245<br><0.001<br>0.190; 0.297**	**R=0.251<br><0.001<br>0.197; 0.304**	R=0.019<br>0.493<br>-0.036; 0.076
Dairy prod-ucts	-	-	**R=0.127<br><0.001<br>0.070; 0.182**	**R=0.114<br><0.001<br>0.057; 0.171**	**R=0.134<br><0.001<br>0.078; 0.189**	R=0.037<br>0.190<br>-0.018; 0.09	**R=0.097<br><0.001<br>0.040; 0.153**	R=0.020<br>0.491<br>-0.036; 0.076	**R=0.066<br>0.021<br>0.010; 0.123**	**R=0.069<br>0.015<br>0.013; 0.126**	R=0.017<br>0.560<br>-0.039; 0.073

Sugar foods	-	-	-	**R=0.071**<br>**0.013**<br>**0.015; 0.128**	**R=0.073**<br>**0.011**<br>**0.016; 0.129**	R=0.035<br>0.226<br>-0.021; 0.091	**R=0.226**<br>**<0.001**<br>**0.171; 0.279**	**R=0.114**<br>**<0.001**<br>**0.057; 0.169**	**R=0.086**<br>**0.003**<br>**0.029; 0.142**	**R=0.109**<br>**<0.001**<br>**0.052; 0.164**	R=0.040<br>0.167<br>-0.016; 0.096
Fruits	-	-	-	-	**R=0.225**<br>**<0.001**<br>**0.170; 0.278**	**R=0.171**<br>**<0.001**<br>**0.115; 0.225**	**R=0.122**<br>**<0.001**<br>**0.065; 0.177**	R=-0.011<br>0.710<br>-0.067; 0.046	**R=0.093**<br>**0.001**<br>**0.037; 0.1499**	R=0.007<br>0.797<br>-0.049; 0.064	R=-0.004<br>0.874<br>-0.061; 0.052
Vegetables	-	-	-	-	-	**R=0.283**<br>**<0.001**<br>**0.230; 0.334**	**R=0.203**<br>**<0.001**<br>**0.148; 0.256**	**R=0.068**<br>**0.017**<br>**0.012; 0.125**	**R=0.278**<br>**<0.001**<br>**0.225; 0.330**	**R=0.172**<br>**<0.001**<br>**0.116; 0.226**	R=-0.001<br>0.946<br>-0.058; 0.054
Legumes	-	-	-	-	-	-	**R=0.198**<br>**<0.001**<br>**0.143; 0.252**	**R=0.068**<br>**0.018**<br>**0.011; 0.124**	**R=0.149**<br>**<0.001**<br>**0.093; 0.204**	**R=0.059**<br>**0.040**<br>**0.002; 0.115**	R=0.044<br>0.124<br>-0.012; 0.101
Grains	-	-	-	-	-	-	-	**R=0.114**<br>**<0.001**<br>**0.058; 0.170**	**R=0.077**<br>**0.007**<br>**0.020; 0.133**	**R=0.091**<br>**0.002**<br>**0.035; 0.147**	R=0.025<br>0.373<br>-0.031; 0.082
Fried foods	-	-	-	-	-	-	-	-	R=-0.016<br>0.579<br>-0.072; 0.040	**R=0.109**<br>**<0.001**<br>**0.053; 0.165**	R=-0.009<br>0.756<br>-0.065; 0.047
Fish	-	-	-	-	-	-	-	-	-	**R=0.145**<br>**<0.001**<br>**0.089; 0.200**	R=-0.026<br>0.364<br>-0.082; 0.030
Eggs	-	-	-	-	-	-	-	-	-	-	R=-0.022<br>0.441

											-0.079; 0.034

Note. R: Pearson correlation coefficient; MD: Mean Difference; P: P-value; 95%CI: 95% Confidence Interval. In **bold** the significant results ($p<0.05$), in *italic* the suggestive ones ($0.05<p<0.10$).

Table S3. "grains" food group profiling by quartiles stratification on red meat intake.

	**Red meat intake (g/day) (Overall, n=1192)**					**Red meat intake (g/day) (Males, n=509)**					**Red meat intake (g/day) (Females, n=683)**				
**"Grains" foods**	**1st q [0-23.1] (n=298)**	**2nd q (23.1-38.3] (n=298)**	**3rd q (38.3- 59.8] (n=298)**	**4th q (59.8-350] (n=298)**	**P-value**	**1st q [0-26.6] (n=128)**	**2nd q (26.6-47.2] (n=127)**	**3rd q (47.2- 67] (n=127)**	**4th q (67-350] (n=127)**	**P-value**	**1st q [0-21.35] (n=171)**	**2nd q (21.35-35] (n=171)**	**3rd q (35-52.55] (n=170)**	**4th q (52.55-267.7] (n=171)**	**P-value**
Grains (g/day)	158.53±83.612	164.038±81.031	192.284±104.44	219.597±107.922	**<0.001**	178.418±89.686	184.546±98.966	220.12±125.157	231.757±106.76	**<0.001**	145.257±78.223	157.31±80.045	175.048±79.158	197.109±100.019	*0.431*
Pastasciutta (g/day)	22.954±27.52	25.374±28.018	32.198±31.57	43.655±38.536	**<0.001**	30.841±31.359	35.920±33.718	42.28±37.325	55.704±42.402	**<0.001**	17.007±20.688	23.663±30.289	22.231±23.161	31.101±27.653	0.431
Egg pasta (g/day)	1.219±3.502	1.778±3.739	2.721±5.902	4.615±9.654	**<0.001**	1.069±2.619	2.389±4.393	3.826±6.996	6.045±12.228	**<0.001**	1.136±3.771	1.473±3.374	2.222±5.622	3.284±6.467	0.15
Filled pasta (g/day)	7.165±11.432	7.442±8.901	9.662±12.183	11.077±13.462	**<0.001**	6.485±11.764	8.671±10.534	9.203±12.570	10.476±14.749	*0.080*	6.602±9.792	8.332±10.332	8.647±10.018	12.157±13.111	0.771
Rice (g/day)	6.524±7.666	6.680±7.001	8.685±9.035	11.311±11.857	**<0.001**	7.150±8.108	7.077±9.075	8.693±9.766	12.640±14.204	**<0.001**	6.197±7.101	6.463±7.089	8.655±7.801	10.140±9.143	*0.086*
Rice salad (g/day)	0.390±0.840	0.467±0.837	0.580±0.852	0.782±1.722	**<0.001**	0.477±0.910	0.449±0.668	0.676±1.057	0.889±2.122	**0.027**	0.342±0.809	0.448±0.902	0.503±0.739	0.726±1.319	0.315
Pasta in soup (g/day)	1.755±3.264	1.850±3.393	2.484±3.627	3.749±6.184	**<0.001**	1.963±3.657	2.356±4.228	2.735±4.389	4.329±7.519	**0.002**	1.597±2.953	1.626±2.528	2.282±3.668	3.188±4.491	0.202
Polenta (g/day)	0.274±0.627	0.384±0.942	0.549±1.833	0.592±1.587	**0.012**	0.212±0.476	0.577±1.375	0.435±1.010	0.552±1.547	*0.054*	0.291±0.684	0.357±0.776	0.597±2.219	0.574±1.554	0.947
Pizza cut (g/day)	0.952±3.703	0.958±3.799	1.769±5.927	1.365±3.751	*0.071*	1.249±4.838	1.189±2.851	2.285±8.199	1.139±3.739	0.255	0.605±2.358	0.925±2.268	1.550±5.183	1.360±3.660	0.371
Pizza from pizzeria	14.667±17.603	14.608±16.421	16.808±18.428	19.921±23.663	**0.002**	15.074±14.948	16.987±17.813	21.283±27.635	22.048±23.541	**0.026**	14.516±18.171	14.067±17.231	16.012±18.510	14.443±14.634	**<0.001**
Homemade pizza (g/day)	11.819±14.177	13.5±19.892	15.023±18.019	14.338±18.703	0.147	11.857±14.873	10.493±12.248	14.438±13.655	13.538±21.619	0.206	11.963±14.204	13.638±23.377	15.877±19.708	16.459±17.586	0.123
Chips or Salads (g/day)	1.186±2.907	1.636±3.204	1.834±3.485	2.182±3.949	**0.004**	1.687±3.146	1.509±2.421	1.711±3.033	1.914±2.836	0.735	0.930±2.622	1.439±3.021	1.929±4.114	2.554±4.817	**<0.001**

Common Breads (g/day)	16.171±30.699	18.037±31.105	24.960±47.072	24.496±39.374	**0.006**	22.507±38.567	24.268±43.331	27.876±53.113	27.661±46.639	0.737	12.636±25.809	15.763±30.038	21.006±32.306	20.402±32.145	0.741
White Bread (g/day)	21.675±43.567	23.167±41.162	30.782±52.133	38.141±55.883	**<0.001**	28.089±49.946	27.951±43.695	42.285±60.945	40.377±55.666	**0.044**	19.182±39.678	21.053±42.028	25.149±45.698	29.844±51.331	*0.064*
Whole grain Sandwiches (g/day)	13.581±25.948	14.828±34.258	10.708±21.380	8.346±18.980	**0.01**	12.149±21.989	12.893±35.369	9.987±21.111	3.801±8.679	**0.009**	14.797±28.611	12.130±24.186	14.422±31.764	12.539±23.462	0.999
Whole grain Crackers (g/day)	1.382±4.170	1.400±4.632	1.736±5.394	1.484±4.505	0.781	1.611±5.220	1.718±4.727	2.040±7.170	0.702±2.474	0.198	1.219±3.269	1.101±4.398	1.729±4.709	1.902±4.633	*0.075*
Whole Bread (g/day)	13.027±31.548	8.289±22.267	7.060±17.039	7.916±24.483	**0.012**	14.183±40.422	8.306±18.682	7.644±20.582	7.785±26.006	0.179	10.153±22.160	10.310±24.998	7.375±16.884	7.208±21.716	0.687
Conditioned breads	4.394±10.658	6.629±14.451	9.403±18.537	12.121±27.930	**<0.001**	6.230±14.409	9.429±16.866	12.032±21.996	13.413±35.215	*0.075*	3.288±8.012	5.716±12.487	6.692±13.655	10.498±21.523	0.605
Toasts (g/day)	8.127±11.467	6.771±11.780	6.184±9.350	4.424±8.963	**<0.001**	8.020±12.384	5.277±9.360	4.557±9.167	3.158±7.734	**<0.001**	8.589±10.961	6.946±12.637	7.281±9.785	6.023±9.855	0.715
Corn Flakes (g/day)	2.714±8.375	3.052±8.783	3.271±9.479	2.732±8.515	0.841	2.815±9.277	2.221±7.821	1.824±7.551	1.789±6.934	0.71	3.140±8.709	3.471±9.409	3.751±9.747	3.728±9.545	0.974
Muesli Oatmeal Fritters (g/day)	2.462±7.853	1.507±6.228	1.126±5.018	1.328±5.713	**0.048**	0.882±4.663	0.920±4.511	0.602±4.119	1.033±4.774	0.891	3.366±9.146	2.144±7.699	1.528±5.441	1.607±6.331	0.481
Whole Grains (g/day)	5.600±10.491	5.445±10.237	4.556±9.008	4.869±12.439	0.594	3.707±8.710	3.719±8.623	3.602±8.198	2.702±8.162	0.732	7.008±11.427	5.941±10.779	5.421±9.167	7.119±15.166	**<0.001**
Seeds (g/day)	0.485±1.208	0.227±0.812	0.175±0.601	0.142±0.602	**<0.001**	0.154±0.565	0.218±0.846	0.096±0.419	0.055±0.288	0.114	0.685±1.441	0.299±0.944	0.180±0.543	0.242±0.789	**0.042**
**Outcome**															
MASLD (yes/no)	148/150 (49.7%/50.3%)	158/140 (53%/47%)	131/167 (44%/56%)	150/148 (50.3%/49.7%)	0.158	68/60 (53.1%/46.9%)	76/51 (59.8%/40.2%)	64/63 (50.4%/49.6%)	70/57 (55.1%/44.9%)	0.486	84/87 (49.1%/50.9%)	80/91 (46.8%/53.2%)	69/101 (40.6%/59.4%)	76/95 (44.4%/55.6%)	0.434

Note. Descriptive statistics are shown as mean±standard deviation or frequencies, as appropriate. q: quartile; n: sample size. In **bold** significant results ($p<0.05$), in *italics* the suggestive ones ($0.05<p<0.10$). MASLD: Metabolic dysfunction-associated steatotic liver disease.

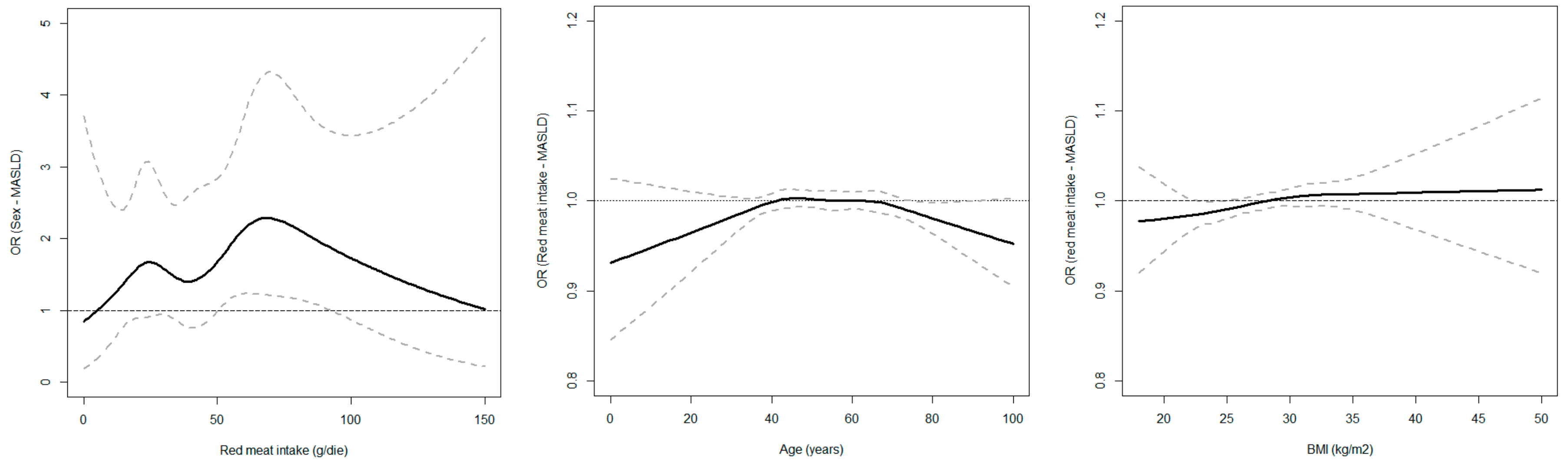

Figure S1. Interaction effects of sex, age and BMI on the relationship between red meat intake and odds ratio (OR) of MASLD

In black is shown the dose response effect, whereas the 95% confidence bounds are presented in dashed gray. ORs were significant by whether their 95% confidence bounds did not involve the value “1”.

OR: odds ratio. MASLD: metabolic dysfunction-associated steatotic liver disease.

The models are adjusted for the potential confounders included in the DAG-related minimal sufficient adjustment set.

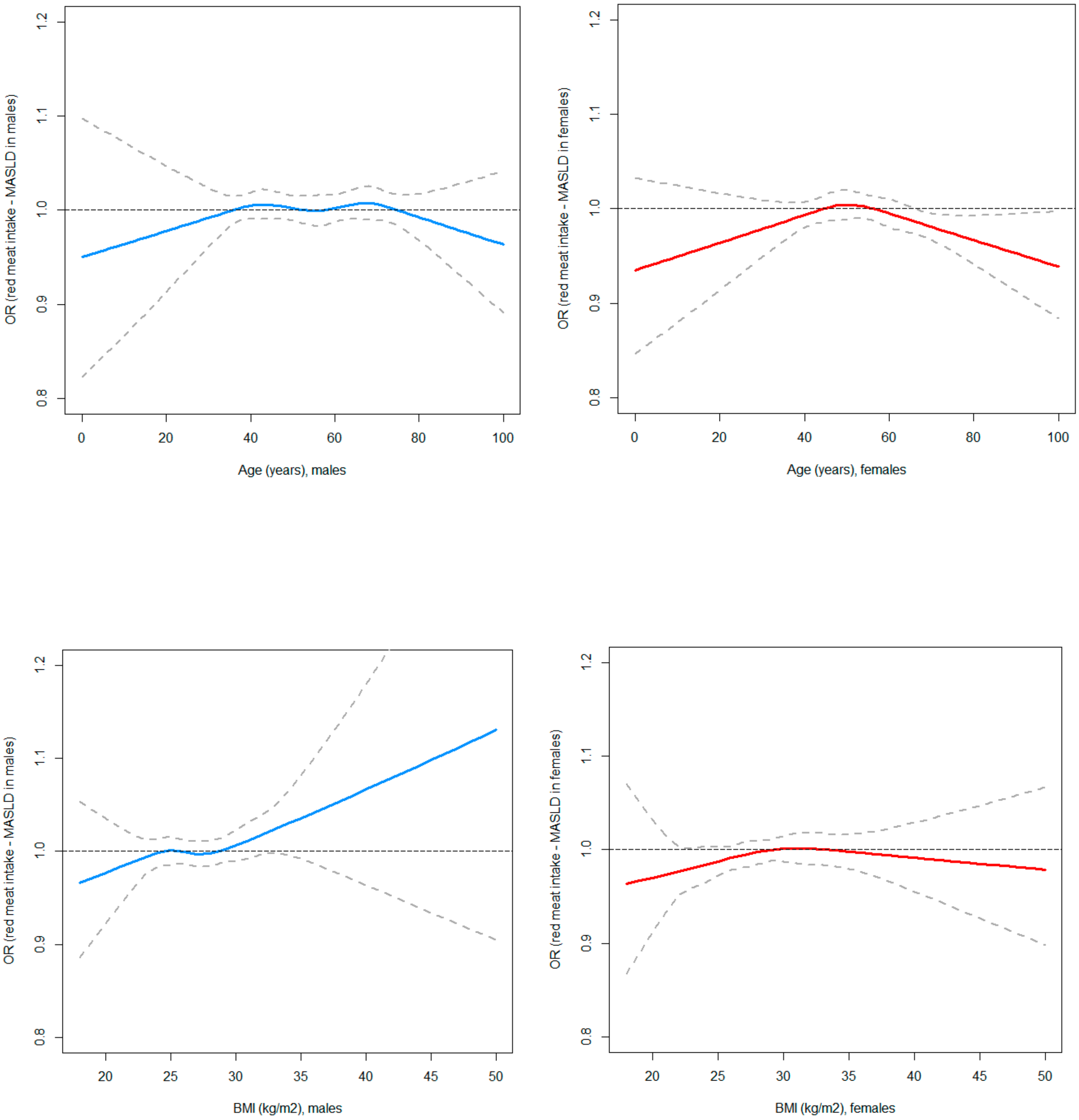

Figure S2. Interaction effects of age and BMI on the relationship between red meat intake and odds ratio (OR) of MASLD, stratified by sex. In cyan and red are shown the dose response effects, whereas the 95% confidence bounds are presented in dashed gray. ORs were significant by whether its 95% confidence bounds did not involve the value “1”: OR: odds ratio. MASLD: metabolic dysfunction-associated steatotic liver disease. The models are adjusted for the potential confounders included in the DAG-related minimal sufficient adjustment set.

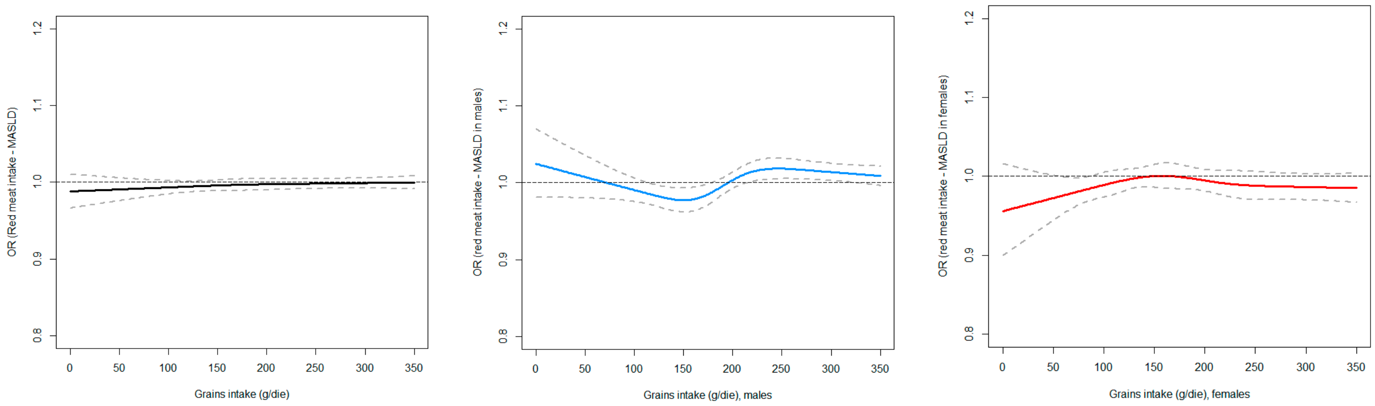

Figure S3. Interaction effects of "grains" group variable on the relationship between red meat intake and odds ratio of MASLD, stratified by sex In black, cyan, and red are shown the dose response effects, whereas the 95% confidence bounds are presented in dashed gray. ORs were significant by whether its 95% confidence bounds did not involve the value "1": OR: odds ratio. MASLD: metabolic dysfunction-associated steatotic liver disease. The models are adjusted for the potential confounders included in the DAG-related minimal sufficient adjustment set.
